# Integrated Molecular, Clinicopathological and Immune Profiling of Non-Small Cell Lung Cancer: The MONREPOS Hellenic Cooperative Oncology Group (HeCOG) Study (HE_2TR/13)

**DOI:** 10.3390/ijms27198724

**Published:** 2026-09-29

**Authors:** Konstantinos Efthymiadis, Chrysa Soteriou, Vassiliki Kotoula, Kyriaki Papadopoulou, Abraham Pouliakis, Sofia Lampaki, Anna Goussia, Elena Fountzilas, Soultana Meditskou, Mattheos Bobos, Konstantinos Kyritsis, Christos Lemesios, Epaminontas Samantas, Evangelos Sarris, George Pentheroudakis, Dimitrios Bafaloukos, Dimitrios Pectasides, Paris A. Kosmidis, Flora Zagouri, Dionysis Spyratos, Eleni Timotheadou, Prodromos Hytiroglou, Alexia Eliades, George Koumbaris, George Fountzilas, Helena Linardou

**Affiliations:** 1 Department of Medical Oncology, Papageorgiou Hospital, School of Health Sciences, Faculty of Medicine, Aristotle University of Thessaloniki, 56403 Thessaloniki, Greece; timotheadou@gmail.com; 2 Medicover Genetics, 2409 Nicosia, Cyprus; chrysa.soteriou@medicover.com (C.S.); christos.lemesios@medicover.com (C.L.); alexia.eliades@medicover.com (A.E.); george.koumbaris@medicover.com (G.K.); 3 Department of Pathology, School of Health Sciences, Faculty of Medicine, Aristotle University of Thessaloniki, 54124 Thessaloniki, Greece; vkotoula@auth.gr (V.K.); pchytiro@auth.gr (P.H.); 4 Laboratory of Molecular Oncology, Hellenic Foundation for Cancer Research, 54124 Thessaloniki, Greece; kyriakipapadopoulou@hotmail.com (K.P.); mbobos@icloud.com (M.B.); fountzil@auth.gr (G.F.); 5 2nd Department of Pathology, Attikon University Hospital, National and Kapodistrian University of Athens, 12461 Athens, Greece; apouliak@med.uoa.gr; 6 Lung Cancer Oncology Unit, Pulmonary Department, G. Papanikolaou Hospital, Aristotle University of Thessaloniki, 57010 Thessaloniki, Greece; sofialampaki@yahoo.gr; 7 Department of Pathology, Ioannina University Hospital, 45500 Ioannina, Greece; agoussia@uoi.gr; 8 Department of Medical Oncology, St Luke’s Clinic, Panorama, 55236 Thessaloniki, Greece; elenafou@gmail.com; 9 School of Medicine, European University Cyprus, 2404 Engomi, Cyprus; 10 Laboratory of Histology and Embryology, Medical School, Aristotle University of Thessaloniki, 54124 Thessaloniki, Greece; meditskou@gmail.com; 11 Institute of Applied Biosciences, Centre for Research and Technology Hellas, 60361 Thessaloniki, Greece; konstantinoskyritsis@certh.gr; 12 Third Department of Medical Oncology, Agii Anargiri Cancer Hospital, 14564 Athens, Greece; epsam@otenet.gr; 13 Fourth Oncology Department and Comprehensive Clinical Trials Center, Metropolitan Hospital, 18547 Athens, Greece; sarris_v@yahoo.gr (E.S.); linardou@icloud.com (H.L.); 14 Department of Medical Oncology, Medical School, University of Ioannina, 45500 Ioannina, Greece; gpentheroudakis@cosmotemail.gr; 15 First Department of Medical Oncology, Metropolitan Hospital, 18547 Athens, Greece; dbafaloukos@metropolitan-hospital.gr; 16 Oncology Section, Second Department of Internal Medicine, Hippokratio Hospital, 11527 Athens, Greece; pectasid@gmail.com; 17 Second Department of Medical Oncology, Hygeia Hospital, 15123 Athens, Greece; parkosmi@otenet.gr; 18 Department of Clinical Therapeutics, Alexandra Hospital, School of Medicine, National and Kapodistrian University of Athens, 11528 Athens, Greece; florazagouri@yahoo.co.uk; 19 Department of Respiratory Medicine, “G. Papanikolaou” General Hospital of Thessaloniki, 57010 Thessaloniki, Greece; dspyrato@auth.gr; 20 Faculty of Medicine, Aristotle University of Thessaloniki, 54124 Thessaloniki, Greece

**Keywords:** non-small cell lung cancer, molecular profiling, tumour-infiltrating lymphocytes, PD-L1, co-mutations, prognosis

## Abstract

Integrated assessment of tumour genomics and immune biomarkers may improve prognostic stratification in non-small cell lung cancer (NSCLC). We examined the distribution and prognostic relevance of somatic pathogenic/likely pathogenic variants (PVs), somatic copy-number alterations (SCNAs), programmed death-ligand 1 (PD-L1) expression and tumour-infiltrating lymphocytes (TILs) in a multicentre cohort. This retrospective, multicentre study included 367 unselected patients with histologically confirmed NSCLC treated at 19 centres in Greece between 2000 and 2020. Targeted next-generation sequencing (NGS) was performed in 267 tumours; PD-L1 tumour proportion score (TPS) and haematoxylin and eosin (H&E)-based TIL density were evaluated in 298 and 352 tumours, respectively. Overall survival (OS) was assessed using Kaplan–Meier and multivariable Cox regression analyses. *TP53* (53.6%) and *KRAS* (16.5%) were the most frequently detected genes with PVs; SCNAs were identified in 22.5% of sequenced tumours and were more frequent in squamous than non-squamous histology. In multivariable analysis (254 complete cases), TIL density ≥10% (hazard ratio [HR] 0.72, 95% confidence interval [CI] 0.52–0.99; *p* = 0.042) and ≥2 mutated genes (HR 1.44, 95% CI 1.08–1.92; *p* = 0.014) were independently associated with OS. *PIK3CA*-containing copy-number gains were significantly enriched in squamous tumours with low TIL density (*p* = 0.024), while *FGFR1*-containing copy-number gains showed a similar but non-significant trend (*p* = 0.088). In the present retrospective study, co-mutation burden and TIL density provided complementary prognostic information beyond clinicopathological variables in NSCLC. The association between selected copy-number gains and an immune-poor squamous phenotype warrants prospective validation.

## 1. Introduction

Lung cancer remains a leading cause of cancer morbidity and mortality worldwide, accounting for 12.4% of all cancer diagnoses and 18.7% of all cancer-related deaths, with 2,480,675 new cases and 1,817,469 deaths recorded in 2021 [1]. Tobacco smoking was initially identified as the primary cause of lung cancer in the 1950s [2,3], and differences in epidemiological patterns reflect different stages of the tobacco epidemic [4], with high-human development index (HDI) countries presenting with higher incidence and mortality rates compared to low-HDI countries [5]. Almost 80–85% of all lung cancer diagnoses today are attributed to smoking; however, several demographic, environmental and biological factors also contribute to disease incidence and outcome variations. Risk factors identified among non-smokers include passive smoking, asbestos exposure, radon exposure, air pollution, and a history of lung cancer in a first-degree relative [6]. Several factors have been linked to inherited genetic predisposition, including germline pathogenic variants (PVs) in DNA-damage response (DDR) genes [7] and oncogenes, such as *EGFR* [8], and *ERBB2* [9]. In addition, genome-wide association studies (GWAS) have revealed chromosomal loci associated with lung cancer susceptibility both in smokers and non-smokers [10].

Non-small cell lung cancer (NSCLC) is the most prevalent histology type of lung cancer, including lung adenocarcinoma (LUAD), lung squamous cell carcinoma (LUSC), and less common large-cell (LCLC) or unclassified histologies. LUAD and LUSC differ in population distribution and have distinct pathological and molecular features [11,12]. Disease stage remains the primary determinant of survival, but outcomes have been reshaped by targeted therapies for driver actionable genomic alterations (AGAs) and immune checkpoint inhibitors (ICIs) for eligible patients [13,14]. Genomic studies have demonstrated that LUAD frequently harbours alterations in RTK/RAS/RAF signalling, whereas LUSC more commonly displays alterations in TP53, cell-cycle regulation, PI3K/AKT signalling, squamous differentiation, and oxidative stress pathways. Co-occurring alterations and somatic copy number alterations (SCNAs) may provide additional biological and prognostic information within driver-defined subgroups [15,16,17,18,19,20].

Programmed Death-Ligand 1 (PD-L1) is a glycoprotein of the B7 membrane protein family (B7-H1), expressed on tumour cells and tumour-infiltrating immune cells, that mediates immune cell evasion primarily through binding to the inhibitory Programmed Death-1 (PD-1) receptors on activated T and B lymphocytes, thereby suppressing anti-tumour immunity [21,22]. PD-L1 tumour proportion score (TPS), evaluated by immunohistochemistry (IHC), is an established predictive biomarker for guiding immuno-oncology (IO)-based systemic anti-cancer therapy (SACT) [23], with higher PD-L1 expression associated with improved treatment outcomes with anti-PD1-/anti-PD-L1- containing regimens in the adjuvant [24], perioperative [25], advanced [26], and metastatic [27] disease settings. Regarding the prognostic role of PD-L1 expression in NSCLC, despite initial contradictory results from retrospective studies, several meta-analyses have reported PD-L1 as a negative prognostic marker, with higher PD-L1 expression associated with poor prognosis [28,29,30], particularly in patients with resected disease [31,32,33].

Tumour-infiltrating lymphocytes (TILs) comprise an integral part of the immune contexture in human cancers, with anti-tumour immune response driven by the patterns of immune cellular infiltrates [34]. In this context, the significance of TIL density in the tumour microenvironment as a positive prognostic biomarker has been established in various solid tumours [35,36]. In patients with NSCLC, higher frequencies of TILs, especially stromal CD8+ T lymphocytes, are associated with improved survival outcomes [37], and the integration of TIL density evaluated on haematoxylin and eosin (H&E)-stained slides with the standard TNM staging system has been proposed for patients with resected disease [38].

In addition, TIL density serves as an accurate biomarker for the prediction of response to ICI-based SACT, and higher TIL densities have been associated with better treatment outcomes [39]. However, the use of TIL density as a biomarker is limited in clinical practice due to variation in scoring approaches driven by the absence of clinically validated standardised cut-offs following evaluation on H&E slides [40]. Novel approaches assessing digital H&E slides with machine learning techniques have also demonstrated the predictive role of TIL density in patients with NSCLC receiving treatment with ICIs [41].

Moreover, the combined predictive role of PD-L1 and TIL density has also been investigated, with evidence suggesting that combining PD-L1 and TILs can predict treatment outcomes more effectively than either biomarker alone [40]. More integrative approaches, using digital slide image analysis with deep learning algorithms, combine the assessment of pathological and molecular data, including PD-L1 expression, TIL density, and mutational status for prognostic stratification in NSCLC [42]. Large datasets that integrate clinicopathological characteristics, targeted sequencing, and PD-L1 and H&E-based TIL assessment remain limited, and integrated analyses may identify interactions between tumour-intrinsic alterations and the immune microenvironment that contribute to prognosis.

To this end, the MONREPOS study was designed to characterise the clinical, pathological, molecular, and immune-biomarker landscape of unselected patients with NSCLC treated at multiple centres across Greece. We evaluated associations among somatic variants, SCNAs, PD-L1 expression, TIL density, and clinicopathological features, and examined their relationship with overall survival (OS).

## 2. Results

### 2.1. Patient and Tumour Characteristics

A total of 367 patients were included. Baseline patient, tumour, and disease characteristics are shown in Table 1 and Table S1. Median age at diagnosis was 65 years (interquartile range [IQR] 59–70), 302 patients (82.3%) were male, 325 (90.3%) were current or former smokers, and 348 (94.8%) had Eastern Cooperative Oncology Group Performance Status (ECOG PS) 0–1. Non-metastatic disease (stage I–III) was present in 216 patients (59.2%) and stage IV disease in 149 (40.8%).

Lung adenocarcinoma (LUAD) was the most common histology (207/367, 56.4%), followed by lung squamous cell carcinoma (LUSC) (129/367, 35.1%); 238 tumours (64.9%) were classified as non-squamous (NSQ). NSQ histology was more frequent in women than in men (80.0% vs. 61.6%; *p* = 0.005). Grade 3–4 tumours accounted for 179/303 evaluable cases (59.1%) and were more frequent in NSQ than LUSC tumours (65.6% vs. 48.2%; *p* = 0.003). Female patients were more frequently never-smokers than male patients (35.9% vs. 4.1%; *p* < 0.001). Detailed clinicopathological cross-tabulations are provided in Table S2.

### 2.2. Molecular and Immune Biomarkers

**Figure 1:**
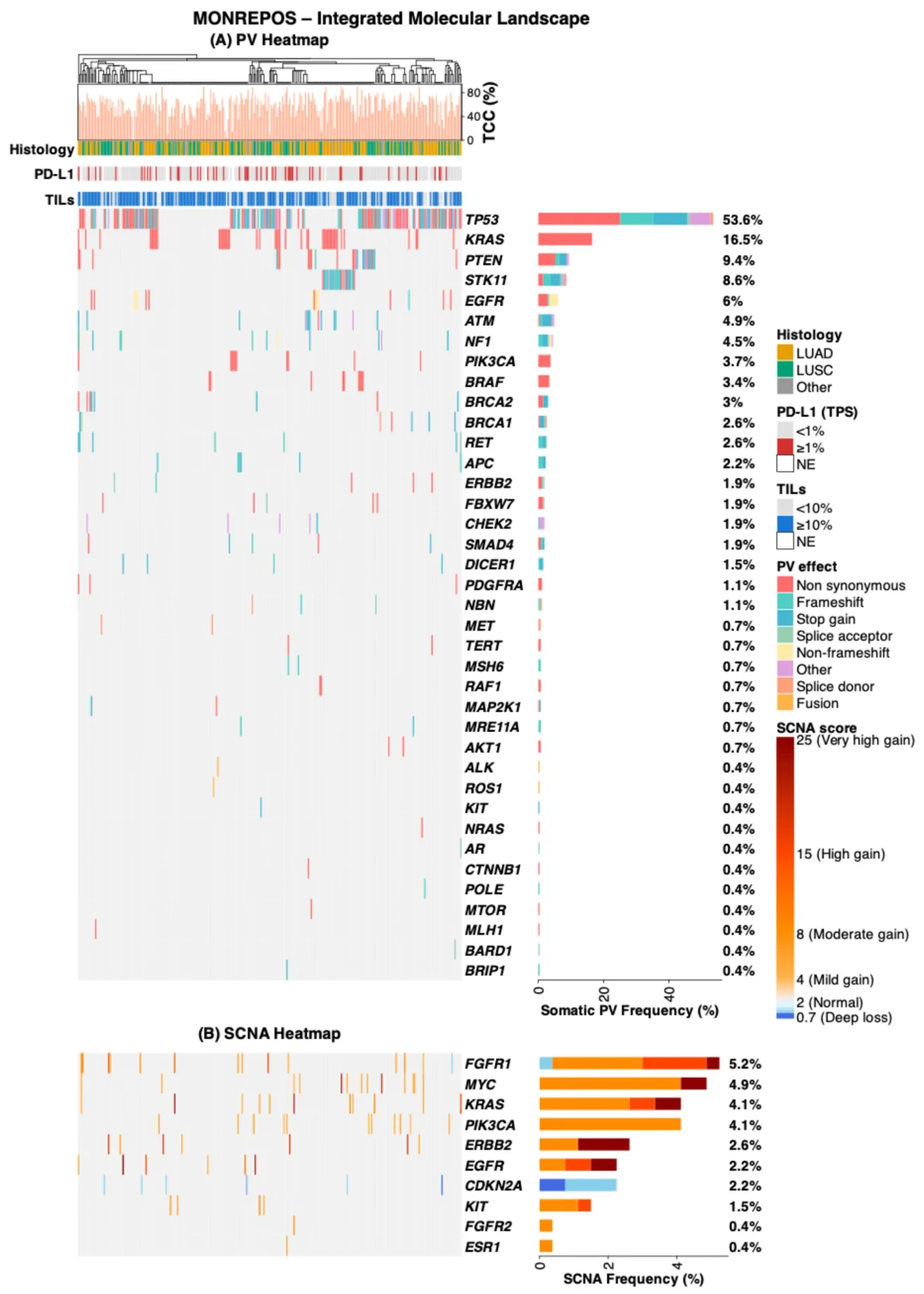
Integrated heatmap of PV/likely PV and SCNA detection, PD-L1 and TIL expression, histology (LUAD/LUSC/Other).

Next-generation sequencing (NGS) was performed in 267 tumours (72.8%); Programmed Death-Ligand 1 (PD-L1) tumour proportion score (TPS) and tumour-infiltrating lymphocyte (TIL) density were available for 298 (81.2%) and 352 (95.9%) tumours, respectively (Table 2). The NGS-sequenced and non-sequenced subcohorts did not differ significantly in any recorded baseline characteristic, and overall survival (OS) was similar between them (median 31.3 vs. 23.4 months; log-rank *p* = 0.172) (Table S3). Detailed biomarker distribution is available in Table S4. Median tumour cell content was 60% (IQR 50–70%). The integrated molecular and immune-biomarker profile is shown in Figure 1.

### 2.3. Somatic Variants and Co-Mutations

#### 2.3.1. Somatic Variants

At least one somatic PV was identified in 218/267 sequenced tumours (81.6%). *TP53* (143/267, 53.6%), *KRAS* (44/267, 16.5%), *PTEN* (25/267, 9.4%), *STK11* (23/267, 8.6%), and *EGFR* (16/267, 6.0%) were the most frequent alterations (Table 2; Figure 1). One, two, three, and ≥4 altered genes were detected in 111 (41.6%), 72 (27.0%), 22 (8.2%), and 13 (4.9%) sequenced tumours, respectively.

**Figure 2:**
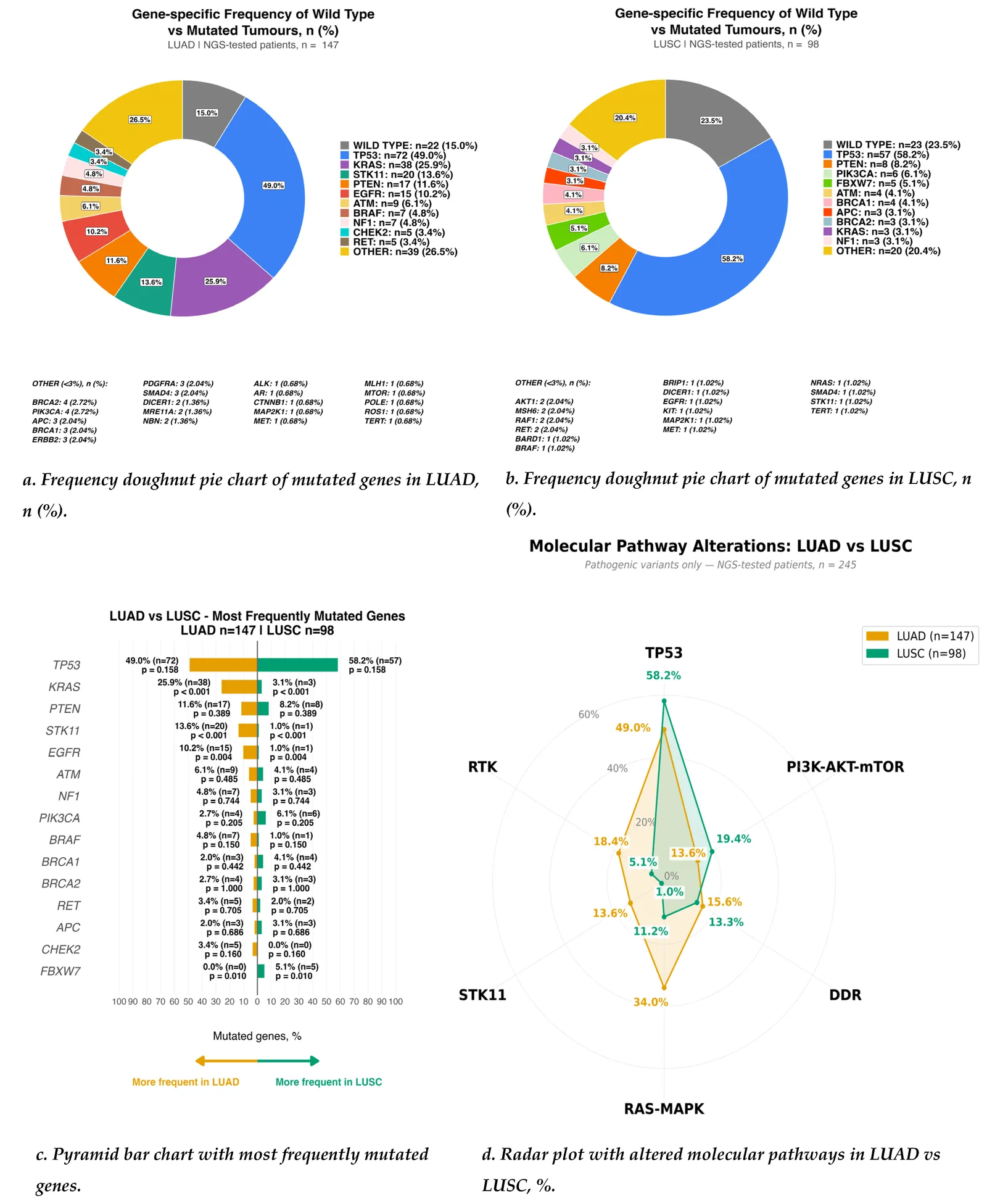
Profiles of detected molecular alterations in LUAD and LUSC; (**a**,**b**): doughnut pie charts, (**c**): pyramid bar chart with statistical testing, (**d**): radar plot. Percentages are calculated per patient; as patients may harbour more than one pathogenic variant, the sum of percentages exceeds 100%.

For LUAD, the most frequent PVs were *TP53* (49.0%), *KRAS* (25.9%), *STK11* (13.6%), *PTEN* (11.6%) and *EGFR* (10.2%). For LUSC, *TP53* (58.2%) predominated, followed by *PTEN* (8.2%), *PIK3CA* (6.1%), *FBXW7* (5.1%) and *ATM* (4.1%). *KRAS*, *STK11,* and *EGFR* alterations were more common in LUAD, whereas *FBXW7* alterations were detected only in LUSC (all *p ≤* 0.010; Figure 2; Table S5).

*TP53* alterations were more common in men than women (57.5% vs. 34.8%; *p* = 0.005). *EGFR* alterations were more frequent in women than men (15.2% vs. 4.1%; *p* = 0.010) and in never-smokers than smokers (31.0% vs. 3.0%; *p* < 0.001). Pathway-level distributions reflected these histology-, sex- and smoking-associated patterns (Figures S1 and S2). PV status comparisons by clinical and tumour characteristics are reported in Table S6.

#### 2.3.2. Co-Mutations

**Figure 3:**
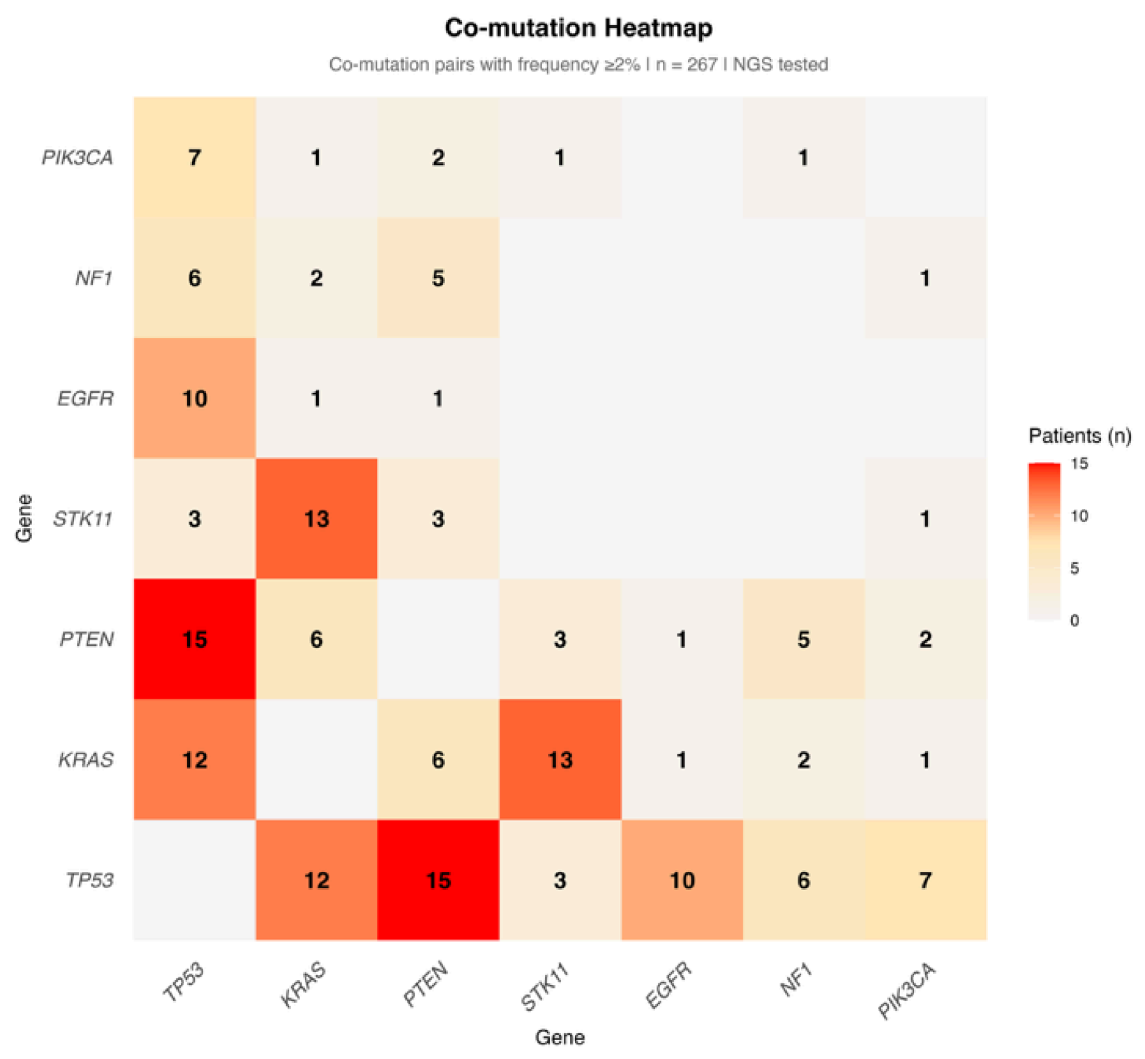
Heatmap of inter-gene pairs involved in co-mutations detected with ≥2% frequency.

At least one co-mutated gene pair was detected in 107/267 tumours (40.1%), and it was more common in NSQ than LUSC tumours (46.2% vs. 29.6%; *p* = 0.008). The most frequent pairs were *PTEN*:*TP53* (5.6%), *KRAS*:*STK11* (4.9%), *KRAS*:*TP53* (4.5%) and *EGFR*:*TP53* (3.7%; Figure 3; Table 3).

Most frequently detected co-mutations varied by tumour histology (NSQ/LUSC), with *KRAS*:*STK11* (7.7% [*n* = 13]) and *PTEN*:*TP53* (6.1% [*n* = 6]) being the most frequently detected co-mutations in tumours with NSQ and LUSC histology, respectively (Table 3). Among male patients, *PTEN*:*TP53* was the most frequently detected co-mutation (6.3% [*n* = 14]), whereas *EGFR*:*TP53* (8.7% [*n* = 4]) was the most frequently detected co-mutation among female patients, regardless of tumour histology (Table S7).

### 2.4. Somatic Copy-Number Alterations (SCNAs)

SCNAs were detected in 60/267 tumours (22.5%) and were more frequent in LUSC than NSQ tumours (31.6% vs. 17.2%; *p* = 0.006). *FGFR1*-containing 8p11.23 gains and *MYC*-containing 8q24.21 gains were each observed in 13 tumours (4.9%). 3q26.32 gains containing *PIK3CA* were confined to LUSC (11.2% vs. 0%; *p* < 0.001), whereas *MYC* gains were more frequent in non-squamous tumours (7.1% vs. 1.0%; *p* = 0.035; Table 4).

### 2.5. PD-L1 Expression

Among 298 tumours assessed for PD-L1, TPS was <1% in 235 (78.9%), 1–49% in 46 (15.4%) and ≥50% in 17 (5.7%); Table S4. Grade 3–4 tumours were more frequent among PD-L1-positive (TPS ≥ 1%) than PD-L1-negative tumours (71.2% vs. 54.7%; *p* = 0.032), as were tumours with TIL density ≥10% (83.6% vs. 70.5%; *p* = 0.040). PD-L1 positive compared to PD-L1 negative tumours were characterised by numerically higher proportions of patients aged < 65 (58.7% vs. 46.8%, *p* = 0.093), male patients (90.5% vs. 80.4%, *p* = 0.062), and smokers (96.8% vs. 88.7%, *p* = 0.056), with none of the aforementioned differences reaching statistical significance. In addition, PD-L1 positivity did not differ by *TP53* mutation status (21.6% of *TP53*-mutant tumours vs. 18.9% of *TP53* wild-type tumours; OR 1.18, 95% CI 0.63–2.22; *p* = 0.599) (Table S8).

**Figure 4:**
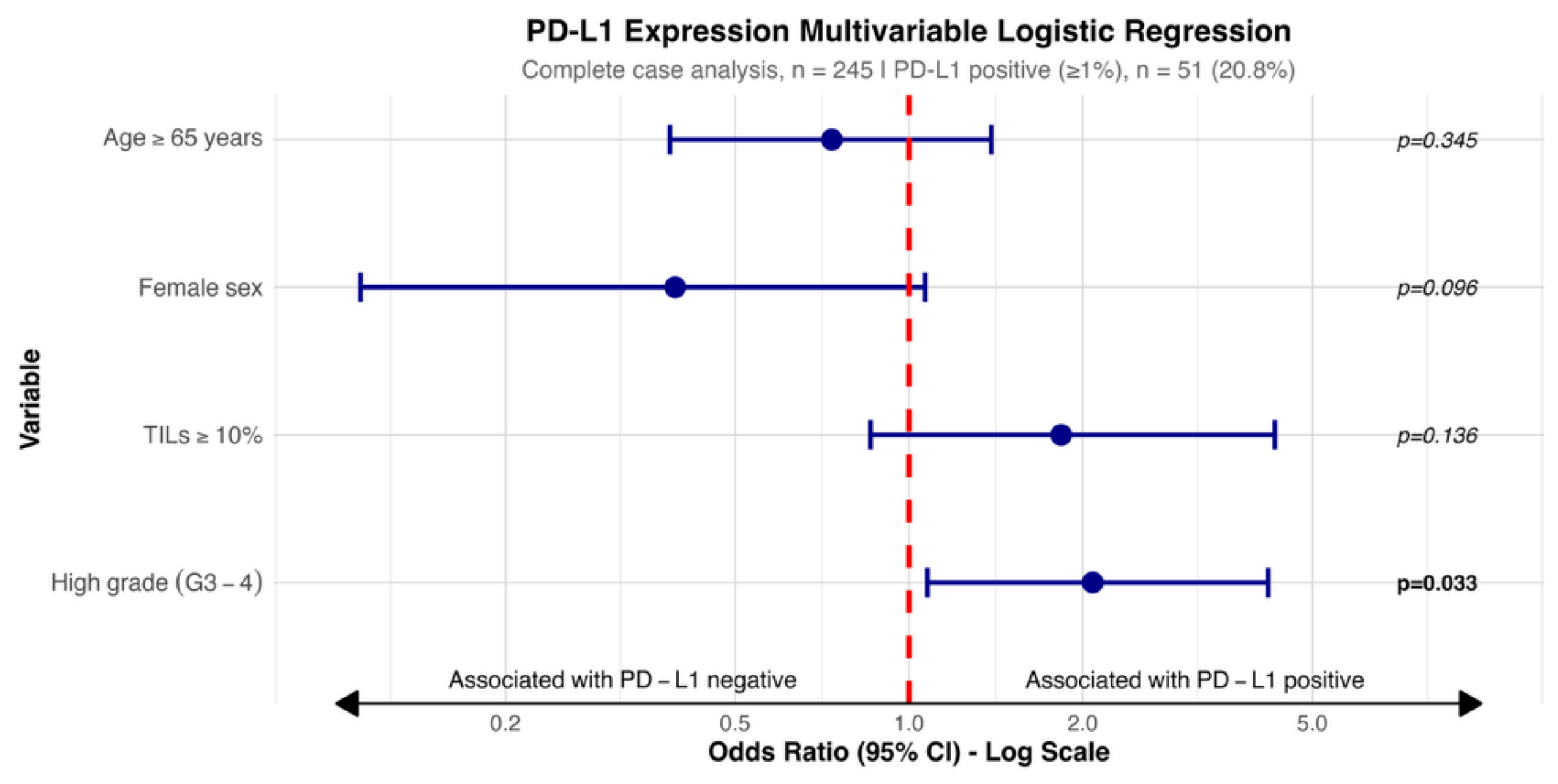
PD-L1 expression multivariable logistic regression forest plot.

In complete-case logistic regression (*n* = 245), high tumour grade was independently associated with PD-L1 positivity (adjusted odds ratio [OR] 2.08, 95% CI 1.08–4.19; *p* = 0.033). Age, sex and TIL density were not independently associated with PD-L1 positivity, although female sex showed a borderline inverse association (OR 0.39, 95% CI: 0.11–1.07; *p* = 0.096). Overall, the model was statistically significant (LR χ^2^(4) = 11.48, *p* = 0.022) (Table 5; Figure 4).

### 2.6. TIL Density

Among 352 tumours assessed for TILs, 254 (72.2%) had TIL density ≥10%, and 133 (37.8%) had TIL density ≥50% (Table 2). TIL density ≥10% was more frequent in stage I–III than stage IV disease (68.0% vs. 41.2%; *p* < 0.001), whereas SCNA-positive tumours were more frequent in the <10% TIL group (30.9% vs. 18.5%; *p* = 0.034). Additionally, TIL density did not differ by *TP53* mutation status (69.9% of *TP53*-mutant tumours vs. 77.7% of *TP53* wild-type tumours; OR 0.67, 95% CI 0.38–1.17; *p* = 0.155) (Table S8).

*PIK3CA*-containing 3q26.32 gains were more frequent in tumours with TIL density <10% than ≥10% (8.8% vs. 2.1%; *p* = 0.024); these gains were confined to LUSC. *FGFR1*-containing 8p11.23 gains showed a similar, non-significant pattern (8.8% vs. 3.2%; *p* = 0.088; Table 6).

### 2.7. Association Between PD-L1 and TIL Expression

**Figure 5:**
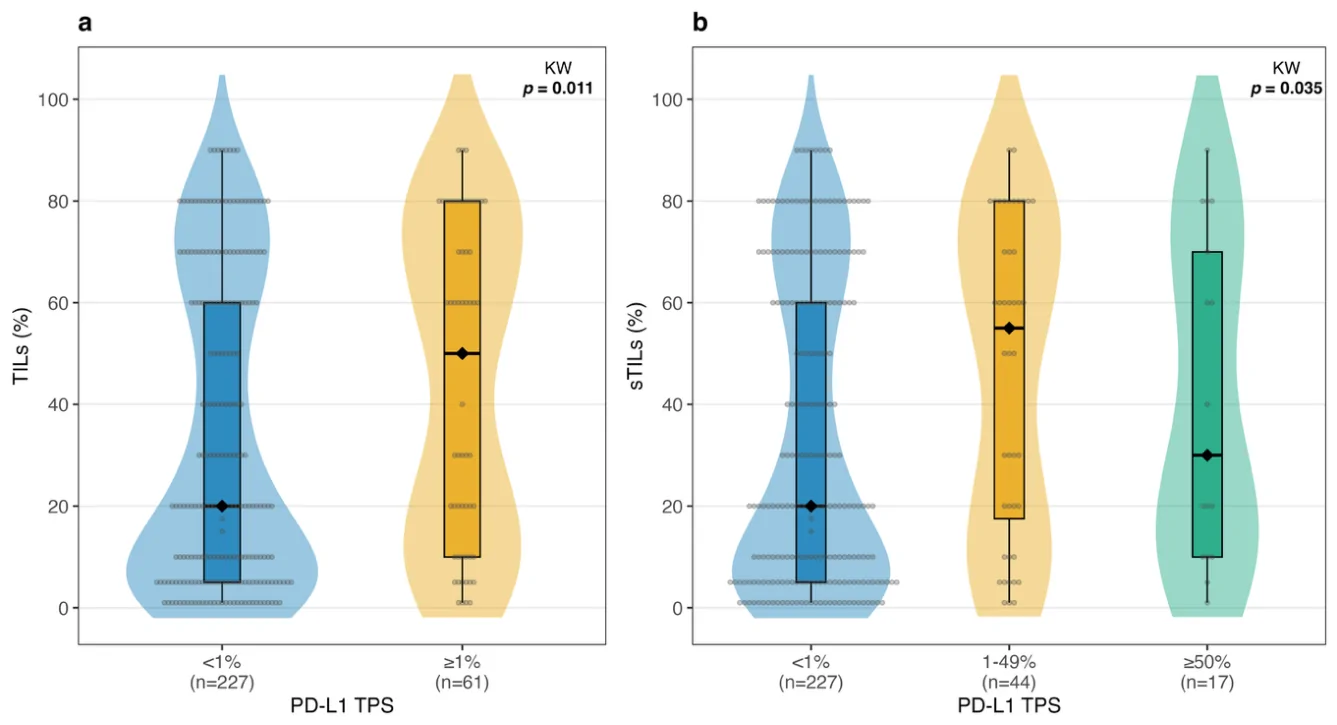
Violin plots with overlaid box plots with *p*-values from the Kruskal–Wallis test, for: (**a**) TILs (%) by PD-L1 expression (<1%/≥1%), (**b**) TILs by PD-L1 expression (<1%/[1–50%)/≥50%).

TIL density was higher in PD-L1-positive than PD-L1-negative tumours (Kruskal–Wallis *p* = 0.011). PD-L1 TPS and TIL expression showed a weak positive monotonic correlation (Spearman r_s_ = 0.146; *p* = 0.013; Figure 5; Table S9).

### 2.8. Treatment Exposure

Treatment exposure is summarised in Table 7. Treatment pathways and drugs prescribed for patients who received systemic anti-cancer treatment (SACT), including treatment with chemotherapy agents, immune checkpoint inhibitors (ICI), and tyrosine kinase inhibitors (TKI), are available in Table S10. Overall, 215 patients (58.6%) underwent surgical resection, and 237 (64.6%) received SACT. ICI-containing SACT was received by 56/237 systemically treated patients (23.6%). Treatment-specific survival comparisons were not undertaken because of stage confounding and major changes in standard treatment across the study period.

### 2.9. Survival Analysis

Survival data were available for 366 patients. Median follow-up was 29.5 months (IQR 12.6–74.5); median OS was 29.5 months (95% CI 25.4–35.0) in the full cohort, 50.3 months (95% CI 40.5–74.5) after resection, and 15.1 months (95% CI 13.2–18.9) from first-line metastatic SACT (Table S11).

Univariable survival analyses with Kaplan–Meier (KM) and log-rank tests are summarised in Table 8 and Figure S3. Older age and ECOG performance status ≥ 2 were associated with shorter OS. Baseline BMI ≥ 25 kg/m^2^ was associated with longer OS in the subset with available data (Table 8). Stage was strongly associated with OS: median OS was 108.8, 49.3, 31.6 and 12.5 months for stages I, II, III and IV, respectively (global log-rank *p* < 0.001). Histology and tumour grade were not associated with OS in univariable analyses (Table 8). TIL density ≥10% was associated with longer OS than TIL density <10% (median 38.6 vs. 16.7 months; univariable HR 0.51, 95% CI 0.39–0.66; *p* < 0.001). PD-L1 TPS ≥ 1% was not associated with OS (*p* = 0.757). Overall PV status and the ≥2-mutated-gene category were not associated with OS in univariable analyses. Individual co-mutation pairs detected in ≥2% of sequenced tumours were likewise not associated with OS (Table S12). For stage IV disease, however, tumours with two or more mutated genes were associated with shorter OS than those with one altered gene or no detected PV (median 9.6, 15.1, and 16.2 months, respectively; *p* = 0.030) (Figure S4). SCNA-positive status was associated with shorter OS in univariable analysis (HR 1.46; *p* = 0.017; Table 8). Treatment-specific OS estimates are not reported because comparisons in this clinically heterogeneous cohort would be substantially confounded by stage, treatment indication and treatment era. Because *TP53* was the most frequently mutated gene, OS was examined within the 143 *TP53*-mutant tumours. Tumours with a PV in *TP53* and in at least one other gene (*n* = 46, 38 deaths) did not differ from *TP53*-only tumours (*n* = 97, 78 deaths) in terms of OS (median 38.0 vs. 30.8 months; HR 1.01, 95% CI 0.68–1.49; *p* = 0.971; adjusted HR 1.08, 95% CI 0.72–1.62; *p* = 0.698), and neither group differed from *TP53* wild-type tumours (global log-rank *p* = 0.974). Two distinct *TP53* PVs within the same tumour were detected in five patients only, and OS was not different from tumours with a single PV in *TP53* (HR 1.47, 95% CI 0.60–3.61; *p* = 0.400) (Table S13).

**Figure 6:**
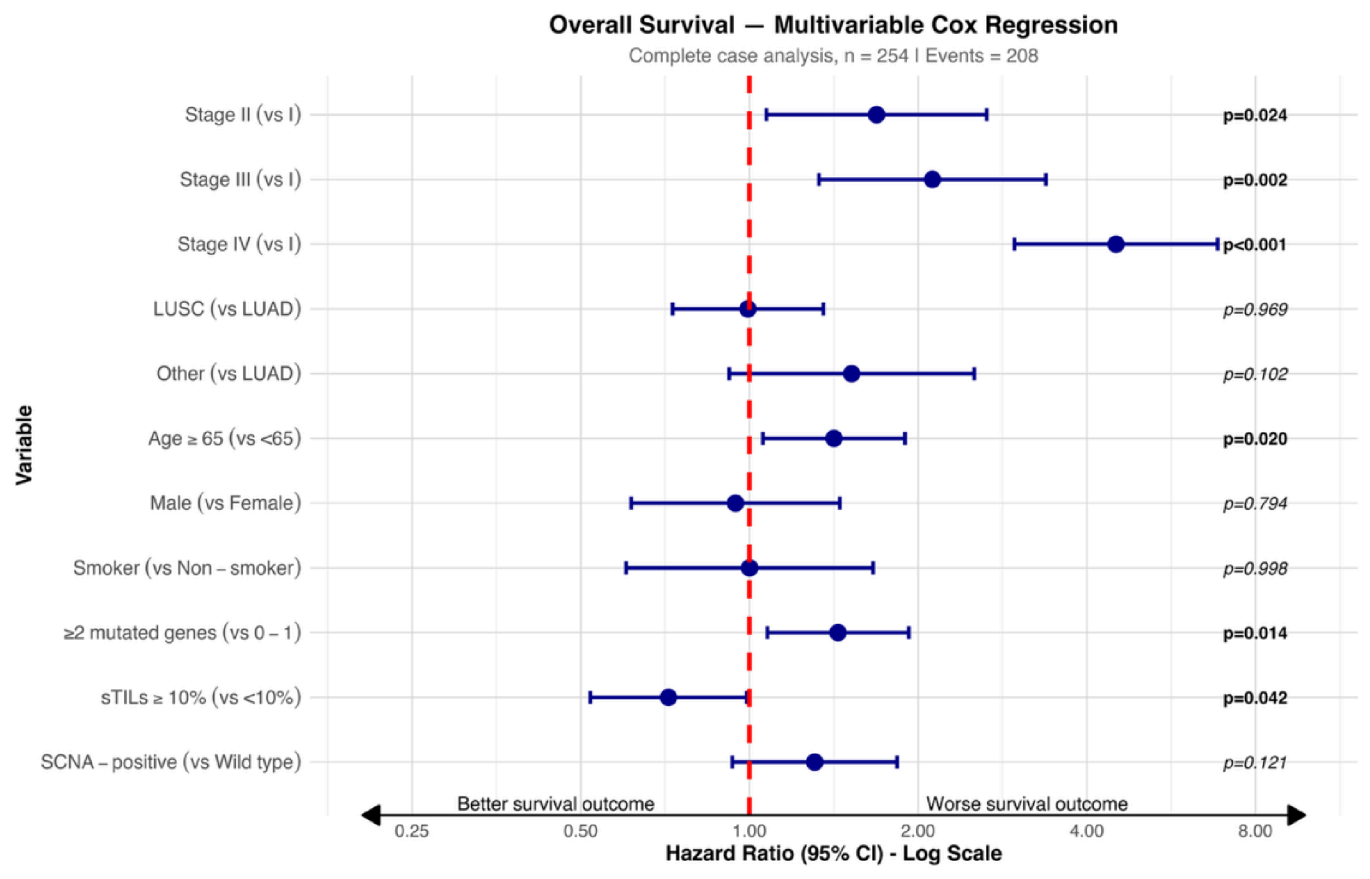
Multivariable Cox Regression forest plot.

A complete-case multivariable Cox model included 254 patients and 208 deaths. The model was significant overall (likelihood-ratio χ^2^ = 90.75; 11 degrees of freedom; *p* < 0.001). After adjustment, age ≥ 65 years (HR 1.42, 95% CI 1.06–1.90; *p* = 0.020), stage II (HR 1.69, 95% CI 1.07–2.65; *p* = 0.024), stage III (HR 2.12, 95% CI 1.33–3.38; *p* = 0.002) and stage IV disease (HR 4.51, 95% CI 2.97–6.85; *p* < 0.001) were associated with shorter OS. TIL density ≥10% was independently associated with longer OS (HR 0.72, 95% CI 0.52–0.99; *p* = 0.042), whereas the presence of two or more mutated genes was associated with shorter OS (HR 1.44, 95% CI 1.08–1.92; *p* = 0.014). Sex, smoking, histology, and SCNA status were not independently associated with OS (Table 9; Figure 6). Because age violated the proportional-hazards assumption (Schoenfeld residual *p* = 0.002), an age-stratified sensitivity analysis was performed; estimates for the remaining covariates were materially unchanged (Table S14).

As the cohort comprised both non-metastatic and metastatic disease, the two biomarkers independently associated with OS in the multivariable model were additionally examined within stage I-III and stage IV disease separately (Table S15). For TIL density ≥10%, the adjusted HR was 0.60 (95% CI 0.40–0.89; *p* = 0.012) in non-metastatic and 0.72 (95% CI 0.50–1.04; *p* = 0.080) in metastatic disease, with median OS of 67.6 vs. 30.0 months (log-rank *p* = 0.004) and 15.2 vs. 9.9 months (log-rank *p* = 0.081), respectively. For the presence of ≥2 mutated genes, the adjusted HR was 1.19 (95% CI 0.81–1.74; *p* = 0.379) in non-metastatic, and 1.83 (95% CI 1.19–2.79; *p* = 0.005) in metastatic disease, with median OS of 48.7 vs. 68.1 months (log-rank *p* = 0.383) and 9.6 vs. 15.1 months (log-rank *p* = 0.009), respectively. Neither biomarker showed statistically significant effect modification by stage (TIL density by stage interaction HR 1.05, 95% CI 0.62–1.80, *p* = 0.849; co-mutation by stage interaction HR 1.51, 95% CI 0.86–2.62, *p* = 0.150).

## 3. Discussion

In this retrospective study, we report the integrated clinicopathological and molecular alteration landscape in a multicentre cohort of 367 unselected patients with non-small cell lung cancer (NSCLC) in Greece. Integrated molecular profiling and routine histopathology identified two independent prognostic associations in NSCLC: tumour-infiltrating lymphocyte (TIL) density ≥ 10% was associated with longer overall survival (OS), whereas the presence of two or more altered genes was associated with shorter OS. The observed histology-specific variant and somatic copy number alteration (SCNA) patterns were broadly consistent with established lung adenocarcinoma (LUAD) and squamous lung carcinoma (LUSC) genomic profiles.

*TP53* was the most frequently detected gene with pathogenic variants (PVs) (53.6%) across histologies, while *KRAS* (25.9%), *STK11* (13.6%) and *EGFR* (10.2%) alterations were enriched in LUAD and *FBXW7* (5.1%) alterations predominated in LUSC. The enrichment of *EGFR* alterations in women and never-smokers is consistent with established clinicogenomic associations. The frequencies of detected *EGFR* mutations were relatively low compared to a previously recorded frequency of 14.9% in LUAD histology in Greek patients with NSCLC [43] in a cohort comprising 51.1% smokers, as opposed to 90.3% smokers and the inclusion of squamous tumours in the present study.

Co-mutation status was the only molecular variable to independently predict shorter OS in multivariable Cox regression (HR 1.44, 95% CI: 1.08–1.92; *p* = 0.014). The absence of a univariable signal (HR 1.19; *p* = 0.198) indicates this association was masked in unadjusted analysis and emerged only after accounting for stage and other clinicopathological factors. The adverse impact was most pronounced in stage IV disease, where patients with two or more mutated genes had an mOS of 9.6 months vs. 15.1 and 16.2 months for patients with one mutated gene and wild-type tumours, respectively (HR 1.87, 95% CI: 1.07–3.26, *p* = 0.030). This association persisted after adjustment (adjusted HR 1.83, 95% CI 1.19–2.79; *p* = 0.005), and the corresponding estimate in non-metastatic disease was 1.19 (95% CI 0.81–1.74; *p* = 0.379). The interaction between co-mutation status and stage did not reach statistical significance (interaction HR 1.51, 95% CI 0.86–2.62; *p* = 0.150). The most prevalent co-mutation pairs involving approved therapeutic options for NSCLC with actionable genetic alterations (AGAs) were *KRAS*:*STK11* [4.9%, enriched in non-squamous tumours (NSQ)] and *EGFR*:*TP53* (3.7%), both carrying documented implications for the guidance of biomarker-directed systemic anti-cancer therapy (SACT) options. *KRAS*:*STK11* co-mutations have been consistently associated with a “cold” tumour microenvironment and subsequent worse outcomes with immune checkpoint inhibitor (ICI)-based regimens [44,45], while *EGFR*:*TP53* co-mutations are associated with shorter progression-free survival on targeted EGFR-TKI SACT monotherapy and identify patients with EGFR mutant tumours who derive greater benefit from the addition of chemotherapy to the targeted agent [46].

The association between the presence of two or more altered genes and shorter OS after multivariable adjustment suggests that aggregate co-mutation burden may capture prognostically relevant genomic complexity. This variable did not establish a treatment-predictive role for any individual co-mutated pair. Consistent with this, none of the individual co-mutated gene pairs detected in ≥2% of sequenced tumours was associated with OS (Table S12), including the pairs discussed above, whose prognostic and predictive relevance is reported in treatment-selected patient populations; with 6 to 15 tumours per pair, these data support the aggregate measure, rather than inference about specific combinations. Furthermore, the stage IV subgroup and stage-stratified findings are exploratory, were not adjusted for multiplicity, and should be interpreted cautiously because of the limited number of patients in each stratum.

SCNA-status was associated with shorter OS in univariable analysis, but not after multivariable adjustment. Sequential adjustment indicated that this attenuation was attributable to stage, as the estimate was essentially unchanged after adjustment for age, sex, smoking status, and histology, and fell when stage was added, with little further change on adjustment for co-mutation status and TIL density. The adjusted estimate remains compatible with a modest adverse effect, and an independent prognostic contribution of copy-number burden cannot be excluded. In addition, SCNAs were enriched in LUSC, particularly *PIK3CA*-containing 3q26.32 and *FGFR1*-containing 8p11.23 gains. Their association with low TIL density is hypothesis-generating: it was based on unadjusted analyses, and SCNA calling is subject to tumour purity-related limitations. Moreover, the *PIK3CA*-low TIL association was statistically significant, whereas the *FGFR1*-low TIL association did not reach statistical significance and should be interpreted as a trend, rather than as a confirmed finding. These findings identify a molecularly defined, immunologically “cold” LUSC subgroup, one characterised by co-occurring *PIK3CA* and/or *FGFR1* copy-number gains and low TIL density, which may warrant further evaluation of combinatorial SCNA-targeting and ICI-based therapeutic approaches.

Programmed death-ligand 1 (PD-L1) tumour proportion score (TPS) positivity (≥1%) was present in only 21.1% of evaluated tumours. This was not related to the stage composition of the cohort, as PD-L1 positivity did not differ between non-metastatic and metastatic disease (*p* = 0.644) (Table S8). No significant association with OS was detected (*p* = 0.757), reinforcing that PD-L1 serves primarily as a predictive rather than prognostic biomarker in an unselected cohort not enriched for ICI receipt. In complete case multivariable logistic regression (*n* = 245), high tumour grade (G3–4) was the sole independent predictor of PD-L1 positivity (adjusted OR 2.08, 95% CI: 1.08–4.19; *p* = 0.033), a finding which is consistent with existing literature [47,48]. A positive but non-significant association of tumours with TILs ≥ 10% with PD-L1 positivity in multivariable analysis (OR 1.84; *p* = 0.136) was corroborated by a significant, albeit weak, monotonic correlation between continuous PD-L1 TPS and TIL values (Spearman rs = 0.146; *p* = 0.013).

TIL density ≥ 10% independently predicted favourable OS in multivariable analysis (HR 0.72, 95% CI: 0.52–0.99; *p* = 0.042), despite a significant association between tumours with TILs ≥10% and earlier disease stage (stage I-III within tumours with TIL density ≥ 10%: 68% vs. 41.2% in tumours with TIL density < 10%; *p* < 0.001), confirming that the prognostic benefit of immunologically “hot” tumours is not attributable to stage distribution alone. Stage-stratified estimates were consistent with this (adjusted HR 0.60, 95% CI 0.40–0.89 in non-metastatic and 0.72, 95% CI 0.50–1.04 in metastatic disease), with no evidence that the effect differed by stage (interaction HR 1.05, 95% CI 0.62–1.80; *p* = 0.849). The magnitude of the univariable HR (0.51, 95% CI: 0.39–0.66; *p* < 0.001) is consistent with existing evidence, in which higher TIL density is associated with improved survival outcomes [37], further supporting the prospective integration of haematoxylin and eosin (H&E)-based TIL evaluation with TNM staging for refined stratification, as previously proposed for the resected disease setting [37,38]. Although this supports the prognostic relevance of H&E-based TIL assessment, the score was semi-quantitative, and the <10% threshold requires prospective analytical and clinical validation before routine use.

## 4. Limitations

Several limitations exist in the present study. The retrospective observational design, the use of data collected over two decades with variability in data quality across the study period, and the heterogeneous inclusion of resected and metastatic disease introduce inherent selection and information biases. Stage was recorded as clinical or pathological stage at diagnosis, reducing granularity for stage-specific analyses. NGS was unavailable for 100 tumours; despite the fact that patients with non-sequenced tumours did not differ from the sequenced subcohort in any recorded clinicopathological characteristic or in overall survival (Table S3), unmeasured differences affecting molecular prevalence estimates cannot be excluded. Baseline BMI data were available for only approximately 50% of the whole cohort (49.9% NA, mainly attributed to patients whose treatment involved only surgical resection), as they were recorded solely for patients who received SACT and were therefore not incorporated into the multivariable OS model despite demonstrating a significant univariable association. In addition, therapies changed substantially over the recruitment period, and only 23.6% of patients who received SACT received an immune-checkpoint inhibitor-containing regimen. Therefore, this cohort cannot reliably estimate treatment-specific effects or immunotherapy-specific outcomes. Moreover, matched germline DNA was not available for NGS in this study. Therefore, although population-frequency filtering was used to remove common germline polymorphisms, a rare germline origin cannot be definitively excluded for individual variants.

TIL assessment was performed semi-quantitatively on H&E-stained slides as a percentage estimate of stromal immune infiltration, recorded in 10% increments, in accordance with international guidelines. This approach does not permit immunophenotypic characterisation of TIL subpopulations, and the biological significance of the complete TIL score may differ from that of specific T-cell subsets, such as CD8+ cytotoxic T lymphocytes or FOXP3+ regulatory T cells, which have been individually assessed in prognostic NSCLC studies. Prospective cohorts with standardised molecular and immune profiling are needed to validate the observed prognostic and biomarker associations.

Finally, the study was conducted across 19 sites in Greece, and the predominantly Greek patient population limits the generalizability of findings to ethnically and geographically diverse NSCLC cohorts. These limitations should be considered when interpreting the results.

## 5. Materials and Methods

### 5.1. Study Design and Patients

MONREPOS was a retrospective, multicentre observational cohort study conducted at 19 Hellenic Cooperative Oncology Group (HeCOG)-affiliated centres in Greece. Clinicopathological data were collected for 367 unselected patients with histologically confirmed NSCLC diagnosed between 2000 and 2020 (GEMA-proN/HE_2TR/13; ClinicalTrials.gov NCT06876402). Patients received treatment for non-metastatic or metastatic disease. Informed consent for clinicopathological data and tissue use for research purposes was given by all patients, and the study protocol was approved by the Committee for Bioethics and Ethics of the Medical School of Aristotle University of Thessaloniki, Greece.

### 5.2. NGS and Molecular Analysis

DNA preparation: DNA was extracted from macrodissected formalin-fixed, paraffin-embedded (FFPE) biopsy or resection specimens using the GeneRead DNA FFPE Kit (Qiagen, Hilden, Germany) following the manufacturer’s instructions. Overall, 2 tissue blocks and 5 sections were acquired per patient. Tumour cell content was estimated independently by two board-certified pathologists as the proportion of tumour cells within all cells in the dissected tissue area [49]. FFPE tissue-derived DNA was quantified using the fluorometric-based Qubit dsDNA High Sensitivity assay (Thermo Fisher Scientific, Waltham, MA, USA). Sequencing was not performed where archival tissue was exhausted or where extracted DNA did not meet quality-control thresholds.

Library preparation: Libraries were prepared using a commercially available Integrated DNA Technologies kit (IDT, Coralville, IA, USA), with 60–250 ng of DNA subjected to enzymatic fragmentation at 32 °C for 7 min, followed by adaptor ligation at 20 °C for 20 min and clean-up using magnetic beads. Subsequently, samples were further subjected to indexing polymerase chain reaction (PCR) and final bead-based clean-up. Library quality was assessed using the 4150 Agilent TapeStation system with D1000 ScreenTape (Agilent, Santa Clara, CA, USA).

Library enrichment: DNA enrichment for the genomic regions of interest was carried out with an in-solution hybridisation-based method using target capture sequences (TACS) specifically designed to capture selected loci in the genes of interest. TACS were immobilised on streptavidin-coated magnetic beads for subsequent hybridisation with the DNA libraries based on a previously established protocol [50]. Sequencing was subsequently performed on an Illumina NovaSeq^TM^ 6000 system (Illumina, San Diego, CA, USA).

Somatic pathogenic/likely pathogenic variants (PVs), including single nucleotide variants (SNVs), multinucleotide variants (MNVs), insertions/deletions, and rearrangements, and somatic copy-number alterations (SCNAs), including copy number gains and copy number losses, were assessed with the ForeSENTIA^®^ Pan-Cancer Core assay (Medicover Genetics, Nicosia, Cyprus). The panel interrogates 80 genes, including 69 assessed for small variants, 17 for SCNAs and 9 for rearrangements (Table S16).

### 5.3. Bioinformatics and Variant Interpretation

Sequencing data were de-multiplexed with bcl2fastq (v.2.16.0), and paired-end DNA sequencing reads were processed to remove adapter sequences and poor-quality reads. The remaining sequences were aligned to the human reference genome build 37 (hg19) using the Burrows-Wheeler alignment algorithm [51]. Duplicate read entries were removed, and aligned read files were converted to a binary alignment map (BAM) format [52]. Variant calling was performed using a versatile somatic variant caller (VarDict) [53]. Annotation of variants was performed using the Ensembl variant effect predictor (VEP) [54]. Primary sequencing, alignment, variant calling and assay-level quality control were performed by Medicover Genetics, whereas secondary VCF harmonisation, annotation and study-specific filtering/curation were performed by the study team in collaboration with a bioinformatician.

Sequencing quality was initially assessed by Medicover Genetics using predefined quality-control criteria, including Q30 scores, with a requirement that >80% of sequenced bases achieve Q30 or higher. Sequencing coverage was considered acceptable when >95% of target bases were covered at a depth of ≥50×. At the provider/assay level, eligible small variants required locus coverage >40×, and variant allele frequency (VAF) ≥5% for single-nucleotide variants or ≥10% for multinucleotide variants and indels. Specifically, sequence alterations such as SNVs and indels were evaluated if the variant allele frequency was ≥5% and ≥10%, respectively, with an analytical sensitivity of ≥97% (95% CI: 94.5–98.5%) and an analytical specificity of ≥99.96% (95% CI: 99.9–99.999%).

For small variants, the respective VCF files for the samples were then submitted to a secondary independent bioinformatic analysis. Specifically, VCF files were at first harmonised using bcftools and further annotated using SnpSift with dbSNP, ClinVar and COSMIC v98, and SnpEff (v5.1d) using the GRCh37.75 reference annotation. Given that the cohort included archival FFPE material collected over an extended period, additional stringent variant filtering was performed in order to accept only those with position/locus coverage >100 reads, variant coverage >40 reads, together with the VAF thresholds of >5% for SNVs or >10% for MNVs/indels. Furthermore, variants showing unexpectedly high recurrence across unrelated tumours and/or evidence of sequencing-batch clustering were reviewed further based on VAF, total and variant read depth, sample and batch distribution, specimen date and evidence from population and cancer databases. Recurrent variants showing patterns consistent with technical or batch-related sequencing artefacts were excluded before downstream molecular and statistical analyses.

Eligible variants were then reviewed against ClinVar and the Catalogue of Somatic Mutations in Cancer (COSMIC) databases [55,56]. Benign and likely benign variants were excluded. Protein-altering coding variants and relevant splice-site alterations with either no reported population frequency or a minor allele frequency (MAF) <0.1% in gnomAD, TOPMed and ExAC were considered for further interpretation [57,58,59,60] [_. Variant pathogenicity and clinical significance were assessed using ClinVar together with the AMP/ASCO/CAP framework for somatic variants [61]. Variants classified as pathogenic/likely pathogenic in ClinVar and/or as having strong or potential clinical significance (AMP/ASCO/CAP Tier I or Tier II) were considered for downstream analysis. Where ClinVar interpretation was absent or conflicting, additional evidence from SnpEff (v5.1d), InterVar (v2.2.1), Franklin, VarSome and CancerVar (accessed in February 2026) was reviewed to support variant interpretation [62,63,64,65,66]. Matched germline DNA was not available herein; therefore, the somatic versus germline origin of individual variants could not be definitively established. Population-frequency filtering was used to exclude common germline polymorphisms, although a rare germline origin cannot be excluded for individual variants detected in the tumours of this study.

Rearrangements were called from discordant-pair and split-read evidence after local assembly, realignment and filtering [67,68,69]. Rearrangements were reported when the variant allele frequency was ≥20%, with an analytical sensitivity and specificity of 100% (95% CI: 66–100%) and 100% (95% CI: 72–100%), respectively.

Gene-level SCNAs were identified with an in-house circular binary segmentation pipeline [70]. Copy number variant calls were accepted as gains if the copy number amplification was either ≥4-fold with tumour DNA purity being >30% or ≥6-fold with tumour DNA purity at 20%. Biallelic deletions in the genes tested for SCNAs were evaluated when the tumour fraction was greater than 50%. The assay demonstrated an analytical sensitivity and specificity of 100% (95% CI: 83.9–100%) and 100% (95% CI: 95.3–100%), respectively, for SCNA detection.

### 5.4. PD-L1 Assessment

PD-L1 expression was assessed on FFPE tissue sections obtained from biopsies or surgical specimens using the Dako 22C3 pharmDx diagnostic assay [71]. IHC staining was performed as per manufacturer’s instructions. IHC stains were assessed by two Board-certified Pathologists using optical microscopy in compliance with the manufacturer’s interpretation instructions for NSCLC [72]. More specifically, PD-L1 expression was measured using tumour proportion score (TPS), defined as the percentage of viable tumour cells with partial or complete membrane staining (≥1+) divided by the total count of all viable tumour cells present in the sample, and multiplied by 100.

### 5.5. TIL Assessment

Tumour-infiltrating lymphocyte (TIL) density was evaluated on FFPE tissue sections obtained from biopsies or surgical specimens, following haematoxylin and eosin (H&E) staining as per established protocols. H&E-stained slides were assessed by two Board-certified Pathologists on optical microscopy. Assessment was based on the International Immuno-Oncology Biomarkers Working Group recommendations, adapted for NSCLC [35].

TILs were estimated within the invasive tumour area in stromal (sTILs) and intratumoural (iTILs) compartments. Scores were recorded in 10% increments, with 1% or 5% increments used for values < 10% [73]. Mononuclear stromal cells, including lymphocytes and plasma cells, were counted; granulocytes, alveolar macrophages, necrotic areas and areas showing pure lepidic or intra-alveolar growth without desmoplastic reaction were excluded.

### 5.6. Statistical Analysis

Analyses were performed in R (v4.5.1) [74]; Images for data visualisation were generated with R (v4.5.1) [74] and Python (v3.13.2) [75]. Baseline demographic, clinicopathological, molecular, immune-biomarker and treatment variables were described using the available-case denominator.

Age was analysed continuously and categorically (<65 or ≥65 years, using the ASCO older individual threshold) [76] and performance status was assessed as per Eastern Cooperative Oncology Group Performance Status (ECOG PS) [77]. Stage was recorded as clinical or pathological stage at diagnosis according to the AJCC 8th edition [78]. Body mass index (BMI) was analysed continuously and categorically as per World Health Organisation (WHO) definition [79]; BMI < 18.5 kg/m^2^: underweight, BMI 18.5–24.9 kg/m^2^: normal, BMI ≥ 25kg/m^2^: overweight, BMI ≥ 30 kg/m^2^: obese. PD-L1 TPS was analysed as <1%, 1–49% and ≥50%, and as binary <1%/≥1% and <50%/≥50% variables. TIL density was analysed as <10%, [10–25%), [25–50%) and ≥50%, and using <10%/≥10% and <50%/≥50% cut-offs [80,81]. Overall survival (OS) was calculated from diagnosis to death from any cause or last follow-up. For the resected cohort, OS was calculated from surgery; for patients receiving first-line systemic anticancer therapy (SACT) for metastatic disease, OS was calculated from treatment start. For exploratory pathway analyses, altered genes were grouped as (1) *TP53*; (2) RAS-MAPK (*KRAS*, *BRAF*, *RAF1*, *MAP2K1*, *NRAS* and *NF1*); (3) PI3K-AKT-mTOR (*PIK3CA*, *AKT1*, *MTOR*, *PTEN* and *FBXW7*); (4) DNA-damage response (DDR) (*MLH1*, *MSH6*, *ATM*, *BRCA1*, *BRCA2*, *CHEK2*, *NBN*, *MREA11*, *BARD1*, *BRIP1*, *POLE*); (5) receptor tyrosine kinase (RTK) (*EGFR*, *ALK*, *ROS1*, *RET*, *ERBB2*, *MET*, *PDGFRA*, *KIT*); (6) *STK11*/*LKB1*; (7) WNT/TGF-beta (*APC*, *CTNNB1*, *SMAD4*); or (8) other pathways.

Continuous variables were reported as median (interquartile range [IQR]) and, where informative, mean (standard deviation [SD]); categorical variables were reported as *n* (%). Group comparisons used Pearson’s chi-square (χ^2^) or Fisher’s exact (FET) tests for categorical variables and t tests/analysis of variance or Mann–Whitney U/Kruskal–Wallis tests for continuous variables, as appropriate after assessment of distributional assumptions. OS was estimated using the Kaplan–Meier method and compared using the log-rank test; univariable and multivariable associations were estimated with Cox regression. The complete-case multivariable Cox model included age, sex, smoking status, histology, stage, TIL density (<10%/≥10%), co-mutation status (<2/≥2 mutated genes) and SCNA status. ECOG performance status and baseline body-mass index were excluded because of sparse and/or missing data. Proportional-hazards assumptions were assessed with Schoenfeld residuals; an age-stratified sensitivity model was fitted when this assumption was not met. Exploratory stage-stratified analyses were performed post hoc, with hazard ratios adjusted for age, sex, smoking status, and histology, and effect modification was assessed by adding a biomarker by stage interaction term to the corresponding model fitted on the pooled cohort and tested by likelihood-ratio test. Associations with PD-L1 TPS ≥ 1% were assessed by complete-case logistic regression; smoking was omitted because of quasi-complete separation. The PD-L1–TIL association was assessed with Spearman correlation and Kruskal–Wallis tests. All tests were two-sided with α = 0.05.

## 6. Conclusions

The MONREPOS study demonstrated, in a multicentre retrospective NSCLC patient cohort in Greece, that co-mutation burden and TIL density, specifically TIL density ≥ 10% and the presence of ≥2 altered genes, are independent prognostic determinants of OS. These findings reflect complementary biological mechanisms of tumour genomic complexity and anti-tumour immune activation that are not fully captured by conventional clinicopathological assessment. Stage-stratified analysis yielded estimates in the same direction in non-metastatic and metastatic disease, with no statistical evidence that either effect differed by stage. The prognostic contribution of genomic complexity was captured by aggregate co-mutation burden, rather than by any individual co-mutated gene pair. The univariable association detected between copy number alteration status and OS was largely explained by disease stage and did not persist after adjustment. The association of LUSC-specific *PIK3CA* and *FGFR1* copy number gains with an immune-cold tumour microenvironment may identify a biologically and therapeutically relevant LUSC subgroup, but remains hypothesis-generating and warrants prospective validation.

## Figures and Tables

**Table 1:** Frequency, *n* (%) ^(1)^ of baseline patient, tumour, and disease characteristics, *n* = 367.

Parameter	*N* (%)
Age	
<65 years old	178 (48.5%)
≥65 years old	189 (51.5%)
Sex	
Female	65 (17.7%)
Male	302 (82.3%)
Smoking status	
Non-smoker	35 (9.7%)
Smoker	325 (90.3%)
NA ^(2)^	7 (1.9%)
ECOG PS	
0–1	348 (94.8%)
≥2	19 (5.2%)
BMI baseline category ^(3)^	
<25 (<normal)	60 (32.6%)
≥25 (≥overweight)	124 (67.4%)
NA	183 (49.9%)
Histology	
NSQ	238 (64.9%)
LUAD	207 (56.4%)
LCLC	17 (4.6%)
Mixed	9 (2.5%)
Unclassified	1 (0.3%)
Undifferentiated	4 (1.1%)
LUSC	129 (35.1%)
Histologic grade	
G1–2	124 (40.9%)
G3–4	179 (59.1%)
NA	64 (17.4%)
Disease stage ^(4)^	
I–III (Non-metastatic)	216 (59.2%)
I	82 (22.5%)
II	60 (16.4%)
III	74 (20.3%)
IIIa	45 (12.3%)
IIIb	26 (7.1%)
IIIc	3 (0.8%)
IV (metastatic)	149 (40.8%)
NA	2 (0.5%)
T stage	
T1–T2	163 (62.9%)
T1	69 (26.6%)
T2	94 (36.3%)
T3–T4	96 (37.1%)
T3	59 (22.8%)
T4	37 (14.3%)
NA	108 (29.4%)
N stage	
N negative	125 (47.9%)
N positive	136 (52.1%)
N1	61 (23.4%)
N2	58 (22.2%)
N3	17 (6.5%)
NA	106 (28.9%)

^(1)^ Percentages (%) calculated using valid denominators unless stated; NA use total denominators. Percentages may not sum to exactly 100% due to 1 decimal rounding. ^(2)^ NA corresponds to not available/missing data. ^(3)^ Baseline body mass index (BMI) refers to the first BMI estimation instance. ^(4)^ Stage refers to either clinical stage or pathological stage at diagnosis as per AJCC 8th edition.

**Table 2:** Histologic and molecular biomarkers.

Parameter	*n* (%) ^(1)^
PD-L1 TPS	
Evaluated	298 (81.2%)
TPS < 1% (negative)	235 (78.9%)
TPS ≥ 1% (positive)	63 (21.1%)
NE ^(2)^	69 (18.8%)
TILs	
Evaluated	352 (95.9%)
<10% TILs	98 (27.8%)
≥10% TILs	254 (72.2%)
NE	15 (4.1%)
NGS	
NGS tested	267 (72.8%)
NE	100 (27.2%)
Gene PV status in 267 tumours	
Wild type	49 (18.4%)
Mutated	218 (81.6%)
1 gene	111 (41.6%)
2 genes	72 (27%)
3 genes	22 (8.2%)
4 genes	11 (4.1%)
5 genes	1 (0.4%)
8 genes	1 (0.4%)
Genes with PV in ≥3% of tumours	
*TP53*	143 (53.6%)
*KRAS*	44 (16.5%)
*PTEN*	25 (9.4%)
*STK11*	23 (8.6%)
*EGFR*	16 (6%)
*ATM*	13 (4.9%)
*NF1*	12 (4.5%)
*PIK3CA*	10 (3.7%)
*BRAF*	9 (3.4%)
*BRCA2*	8 (3%)
Co-mutation status in 267 tumours	
<2 mutated genes	160 (59.9%)
≥2 mutated genes	107 (40.1%)
Co-mutations in ≥3% of tumours	
*PTEN/TP53*	15 (5.6%)
*KRAS/STK11*	13 (4.9%)
*KRAS/TP53*	12 (4.5%)
*EGFR/TP53*	10 (3.7%)
SCNA status in 267 tumours	
No SCNA	207 (77.5%)
SCNA-positive	60 (22.5%)
1 SCNA	49 (18.4%)
≥2 SCNAs	11 (4.1%)
SCNAs in ≥3% of tumours	
8p11.23 gain (*FGFR1*)	13 (4.9%)
8q24.21 gain (*MYC*)	13 (4.9%)
12p12.1 gain (*KRAS*)	11 (4.1%)
3q26.32 gain (*PIK3CA*)	11 (4.1%)

^(1)^ Percentages (%) calculated using valid denominators unless stated; NE use total denominators. Percentages may not sum to exactly 100% due to 1 decimal rounding. ^(2)^ NE corresponds to patients whose tissue was not tested.

**Table 3:** Co-mutation detection among 267 NGS-tested tumours.

Co-Mutation Status by Tumour Histology.								
Co-Mutation Status		All, *n* (%)		NSQ, *n* (%)		LUSC, *n* (%)		*p* Value
NGS-tested tumours		267 (100%)		169 (100%)		98 (100%)		
<2 mutated genes		160 (59.9%)		91 (53.8%)		69 (70.4%)		0.008^χ2^
≥2 mutated genes		107 (40.1%)		78 (46.2%)		29 (29.6%)		
Individual co-mutations with ≥2% frequency by tumour histology.								
All histologies, *n* = 267			NSQ, *n* = 169				LUSC, *n* = 98	
Co-mutation	*n* (%)		Co-mutation		*n* (%)		Co-mutation	*n* (%)
*PTEN/TP53*	15 (5.6%)		*KRAS/STK11*		13 (7.7%)		*PTEN/TP53*	6 (6.1%)
*KRAS/STK11*	13 (4.9%)		*KRAS/TP53*		11 (6.5%)		*PIK3CA/TP53*	5 (5.1%)
*KRAS/TP53*	12 (4.5%)		*EGFR/TP53*		9 (5.3%)		*FBXW7/TP53*	4 (4.1%)
*EGFR/TP53*	10 (3.7%)		*PTEN/TP53*		9 (5.3%)		*APC/TP53*	3 (3.1%)
*PIK3CA/TP53*	7 (2.6%)		*KRAS/PTEN*		6 (3.6%)		*AKT1/TP53*	2 (2%)
*KRAS/PTEN*	6 (2.2%)		*ATM/KRAS*		5 (3%)		*ATM/TP53*	2 (2%)
*NF1/TP53*	6 (2.2%)		*NF1/PTEN*		5 (3%)		*BRCA1/TP53*	2 (2%)

**Table 4:** SCNAs detected among the 267 NGS-tested tumours by tumour histology.

			All Histologies, *n* = 267	NSQ, *n* = 169	LUSC, *n* = 98	
			*n* (%)	*n* (%)	*n* (%)	*p*-Value ^(1)^
SCNA status						
No SCNA			207 (77.5%)	140 (82.8%)	67 (68.4%)	0.006**^χ2^**
SCNA-positive			60 (22.5%)	29 (17.2%)	31 (31.6%)	
Individual SCNA detected						
chr (cytoband)	Gene	Status				
chr8 (8p11.23)	*FGFR1*	Gain	13 (4.9%)	4 (2.4%)	9 (9.2%)	0.018^FET^
chr8 (8q24.21)	*MYC*	Gain	13 (4.9%)	12 (7.1%)	1 (1%)	0.035^FET^
chr12 (12p12.1)	*KRAS*	Gain	11 (4.1%)	8 (4.7%)	3 (3.1%)	0.751^FET^
chr3 (3q26.32)	*PIK3CA*	Gain	11 (4.1%)	0 (0%)	11 (11.2%)	<0.001^FET^
chr17 (17q12)	*ERBB2*	Gain	7 (2.6%)	3 (1.8%)	4 (4.1%)	0.265^FET^
chr9 (9p21.3)	*CDKN2A*	Loss	6 (2.2%)	3 (1.8%)	3 (3.1%)	0.672^FET^
chr7 (7p11.2)	*EGFR*	Gain	6 (2.2%)	5 (3%)	1 (1%)	0.419^FET^
chr4 (4q12)	*KIT*	Gain	4 (1.5%)	2 (1.2%)	2 (2%)	0.626^FET^
chr6 (6q25.1)	*ESR1*	Gain	1 (0.4%)	0 (0%)	1 (1%)	0.367^FET^
chr8 (8p11.23)	*FGFR1*	Loss	1 (0.4%)	1 (0.6%)	0 (0%)	1.000^FET^
chr10 (10q26.13)	*FGFR2*	Gain	1 (0.4%)	1 (0.6%)	0 (0%)	1.000^FET^

^(1)^ Statistical testing performed within the 267 NGS-tested tumours. Pearson’s chi square was used if all expected counts were 5 or more; Fisher’s exact test was used if any expected count was below 5.

**Table 5:** Univariable and multivariable logistic regression for PD-L1 expression (TPS cut-off 1%) ^(1)^.

	Logistic Regression					
	Univariable			Multivariable ^(2)^		
Factor	OR	(95% CI)	*p* Value	OR	(95% CI)	*p* Value
Age; ≥65 years vs. <65 years	0.73	(0.39–1.35)	0.317	0.74	(0.39–1.39)	0.345
Sex; Female vs. Male	0.40	(0.12–1.07)	0.099	0.39	(0.11–1.07)	0.096
Grade; High (G3–4) vs. Low (G1–2)	2.08	(1.09–4.14)	0.031	2.08	(1.08–4.19)	0.033
TILs (%); ≥10% vs. <10%	1.75	(0.83–4.07)	0.161	1.84	(0.86–4.30)	0.136

^(1)^ Complete case analysis, *n* = 245. ^(2)^ Likelihood Ratio Test χ^2^ = 11.48, df = 4, *p* = 0.022.

**Table 6:** SCNAs detected among NGS-tested and TIL density-evaluated tumours, *n* = 257.

NGS Tested, *n* = 267 ^(1)^				Evaluated for TILs (%), *n* = 257			
Individual SCNA Detected				All, *n* = 257 ^(2)^	TILs < 10%, *n* = 68	TILs ≥ 10%, *n* = 189	
chr/Cytoband		Gene	Status	*n* (%)	*n* (%)	*n* (%)	*p*-Value ^(2)^
chr8	8p11.23	*FGFR1*	Gain	12 (4.7%)	6 (8.8%)	6 (3.2%)	0.088^FET^
	8q24.21	*MYC*	Gain	11 (4.3%)	5 (7.4%)	6 (3.2%)	0.166^FET^
chr12	12p12.1	*KRAS*	Gain	10 (3.9%)	2 (2.9%)	8 (4.2%)	1.000^FET^
chr3	3q26.32	*PIK3CA*	Gain	10 (3.9%)	6 (8.8%)	4 (2.1%)	0.024^FET^
chr17	17q12	*ERBB2*	Gain	7 (2.7%)	0 (0%)	7 (3.7%)	0.195^FET^
chr9	9p21.3	*CDKN2A*	Loss	6 (2.3%)	3 (4.4%)	3 (1.6%)	0.191^FET^
chr7	7p11.2	*EGFR*	Gain	5 (1.9%)	2 (2.9%)	3 (1.6%)	0.610^FET^
chr4	4q12	*KIT*	Gain	4 (1.6%)	1 (1.5%)	3 (1.6%)	1.000^FET^
chr6	6q25.1	*ESR1*	Gain	1 (0.4%)	0 (0%)	1 (0.5%)	1.000^FET^
chr8	8p11.23	*FGFR1*	Loss	1 (0.4%)	1 (1.5%)	0 (0%)	0.265^FET^
chr10	10q26.13	*FGFR2*	Gain	1 (0.4%)	0 (0%)	1 (0.5%)	1.000^FET^

^(1)^ NGS performed in 267 patients. ^(2)^ TIL expression evaluated in 257 NGS-tested tumours. Percentages are calculated within columns. Statistical testing performed within NGS-tested and TIL expression-evaluated tumours (*n* = 257).

**Table 7:** Treatment modalities.

Parameter	*n* (%)
Type of treatment	
Any type of treatment	
Yes	340 (92.6%)
No	27 (7.4%)
Surgical resection	
Yes	215 (58.6%)
No	152 (41.4%)
Type of surgery in 215 patients	
Lobectomy	151 (70.2%)
Pneumonectomy	38 (17.7%)
Segmentectomy	16 (7.4%)
Other	6 (2.8%)
NA	4 (1.9%)
CCRT	
Yes	6 (1.6%)
No	361 (98.4%)
SACT ^(1)^	
Yes	237 (64.6%)
No	130 (35.4%)
Lines of SACT	
Neoadjuvant SACT	
Yes	6 (1.6%)
No	361 (98.4%)
Adjuvant SACT	
Yes	58 (15.8%)
No	309 (84.2%)
1st line SACT	
Yes	202 (55%)
No	159 (45%)
2nd line SACT	
Yes	112 (30.5%)
No	252 (69.5%)
≥3rd line SACT	
Yes	41 (11.2%)
No	326 (88.8%)
ICI- and TKI-based SACT ^(2)^	
ICI-based SACT any line	*n* = 237
Yes	56 (23.6%)
No	191 (76.4%)
ICI-based 1st-line SACT	*n* = 202
Yes	17 (8.4%)
No	185 (91.6%)
ICI-based 2nd-line SACT	*n* = 112
Yes	30 (26.8%)
No	82 (73.2%)
ICI-based ≥ 3rd-line SACT	*n* = 41
Yes	11 (26.8%)
No	30 (73.2%)
TKI-based SACT any line	*n* = 237
Yes	45 (19%)
No	192 (81%)
TKI-based 1st-line SACT	*n* = 202
Yes	22 (10.9%)
No	180 (89.1%)
TKI-based 2nd-line SACT	*n* = 112
Yes	19 (17%)
No	93 (83%)

^(1)^ SACT prescribed as standard of care or within a clinical trial. Treatment pathways and drugs prescribed are available in Table S10. ^(2)^ ICI included Atezolizumab, Durvalumab, Ipilimumab, Nivolumab, and Pembrolizumab. TKI drugs included Afatinib, Cabozantinib, Crizotinib, Erlotinib, Gefitinib, Lapatinib, Nintedanib, and Osimertinib prescribed either for NSCLC with AGAs or as ≥2nd line options for NSCLC with LUSC histology.

**Table 8:** KM survival analysis and Log-rank Test ^(1)^.

			KM Survival Analysis		Log-Rank Test and Hazard Ratio (HR)			
Factor	*n*	Events	OS (Months)	(95% CI)		HR	(95% CI)	Log-Rank *p* Value
Patient characteristics								
Age	366	306			≥65 years	1.7	(1.35–2.15)	<0.001
<65 years	178	139	38.6	(31.5–50.3)	<65 years (ref.)			
≥65 years	188	167	24.2	(20.9–29.3)				
Sex	366				Male	1.12	(0.83–1.50)	0.465
Female	65	54	31.5	(20.6–70.5)	Female (ref.)			
Male	301	252	28.1	(25.0–35.0)				
Smoking status	359	301			Smoker	1.07	(0.73–1.58)	0.7157
Non-smoker	35	29	31.3	(20.4–98.7)	Non-smoker (ref.)			
Smoker	324	272	28.2	(25.3–35.5)				
BMI baseline	179	152			≥Overweight	0.68	(0.48–0.95)	0.023
≤Normal	59	53	20.9	(14.4–27.1)	≤Normal (ref.)			
≥Overweight	120	99	28.7	(20.4–37.0)				
ECOG PS	366	306			ECOG ≥ 2	3.11	(1.94–4.98)	<0.001
0–1	347	287	30.9	(26.5–37.7)	0–1 (ref.)			
≥2	19	19	10.3	(5.1–31.0)				
Tumour characteristics								
Histology	366	306			LUSC	0.96	(0.75–1.22)	0.424
LUAD	206	172	29.6	(23.5–37.0)	Other histology	1.27	(0.85–1.89)	
LUSC	129	106	31.5	(26.5–44.2)	LUAD (ref.)			
Other	31	28	14.4	(12.5–57.1)				
Grade	302	245			G3–4	1.03	(0.80–1.33)	0.800
G1–2	123	104	38.5	(29.6–55.7)	G1–2 (ref.)			
G3–4	179	141	27.4	(21.4–37.0)				
Stage	364	304			Stage II	1.23	(0.83–1.82)	<0.001
Stage I	81	59	108.8	(68.1–131.1)	Stage III	2.13	(1.47–3.09)	
Stage II	60	43	49.3	(34.0–112.8)	Stage IV	4.57	(3.31–6.30)	
Stage III	74	60	31.6	(26.0–48.7)	Stage I (ref.)			
Stage IV	149	142	12.5	(9.4–15.3)				
Microscopy biomarkers								
PD-L1 expression	298	245			TPS ≥ 1%	0.95	(0.69–1.31)	0.757
TPS ≤ 1%	235	198	32.1	(27.0–39.7)	TPS < 1% (ref.)			
TPS > 1%	63	47	30.9	(23.3–88.1)				
TIL density	351	292			TILs ≥ 10%	0.51	(0.39–0.66)	<0.001
TILs < 10%	98	87	16.7	(12.8–21.9)	TILs < 10% (ref.)			
TILs ≥ 10%	253	205	38.6	(31.5–50.3)				
Molecular biomarkers								
Gene PV status	267	219			Mutated	1.07	(0.75–1.51)	0.711
Wild type	49	39	31.5	(18.9–74.5)	Wild type (ref.)			
Mutated	218	180	30.9	(26.2–39.7)				
Co-mutation status	267	219			≥2 mutated genes	1.19	(0.91–1.56)	0.198
<2 mutated genes	160	128	30.6	(25.1–49.8)	<2 mutated genes (ref.)			
≥2 mutated genes	107	91	33.7	(25.4–43.5)				
Co-mutation status (stage IV)	106	100			≥2 mutated genes- stage IV	1.87	(1.07–3.26)	0.030
Wild type	22	19	16.2	(9.4–65.8)	1 mutated gene- stage IV	1.13	(0.65–1.95)	
1 mutated gene	44	41	15.1	(9.9–30.2)	Wild type- stage IV (ref.)			
≥2 mutated genes	40	40	9.6	(6.4–16.3)				
SCNA status	267	219			SCNA-positive	1.46	(1.07–2.00)	0.017
No SCNA detected	207	165	36.6	(28.4–49.3)	No SCNA detected (ref.)			
SCNA-positive	60	54	26.2	(20.6–32.3)				

^(1)^ Statistical testing excluding NA and NE.

**Table 9:** Complete case multivariable Cox regression ^(1)^.

Factor	HR	(95% CI)	*p* Value	*n*
Patient characteristics				
Age: ≥65 years (vs. <65 years)	1.42	(1.06–1.90)	0.020	254
Sex: Male (vs. Female)	0.94	(0.62–1.45)	0.794	254
Smoking status: Smoker (vs. Non-smoker)	1.00	(0.60–1.66)	0.998	254
Tumour characteristics				
Histology: LUSC (vs. LUAD)	0.99	(0.73–1.36)	0.969	254
Histology: Other (vs. LUAD)	1.52	(0.92–2.52)	0.102	254
Stage II (vs. I)	1.69	(1.07–2.65)	0.024	254
Stage III (vs. I)	2.12	(1.33–3.38)	0.002	254
Stage IV (vs. I)	4.51	(2.97–6.85)	<0.001	254
Microscopy biomarkers				
TIL expression: TILs ≥10% (vs. <10%)	0.72	(0.52–0.99)	0.042	254
Molecular biomarkers				
Co-mutation status: ≥2 mutated genes (vs. <2)	1.44	(1.08–1.92)	0.014	254
SCNA status: SCNA-positive (vs. no SCNA detected)	1.31	(0.93–1.83)	0.121	254

^(1)^ Complete case analysis, *n* = 254, events = 208.

## Data Availability

All research-relevant data are within the paper. The complete catalogue of somatic pathogenic variants and somatic copy number alterations detected is provided as Dataset S1. NGS raw data are publicly available at https://doi.org/10.6084/m9.figshare.34013304.

## Supplementary Materials

The following supporting information can be downloaded at: https://www.mdpi.com/article/10.3390/ijms27198724/s1.

## Abbreviations

The following abbreviations are used in this manuscript:

AGA	Actionable Genetic Alteration
AJCC	American Joint Committee on Cancer
AKT	AKT Serine/Threonine Kinase
AMP	Association for Molecular Pathology
ASCO	American Society of Clinical Oncology
B7-H1	B7 Homologue 1
BAM	Binary Alignment Map
BMI	Body Mass Index
CAP	College of American Pathologists
CCRT	Concurrent chemoradiotherapy
CD	Cluster of Differentiation
CI	Confidence Intervals
COSMIC	Catalogue Of Somatic Mutations in Cancer
DDR	DNA-Damage Response
df	Degrees of Freedom
DNA	DeoxyriboNucleic Acid
dsDNA	Double-stranded Deoxyribonucleic Acid
ECOG PS	Eastern Cooperative Oncology Group Performance Status
ExAc	Exome Aggregation Consortium
FET	Fisher’s Exact Test
FFPE	Formalin-Fixed Paraffin Embedded
G1–2	Grade 1–2
G2–3	Grade 2–3
gnomAD	Genome Aggregation Database
GAS	Genome-Wide Association Studies
H&E	Haematoxylin and Eosin
HDI	Human Development Index
HeCOG	Hellenic Cooperative Oncology Group
HR	Hazard Ratio
ICF	Informed Consent Form
ICI	Immune Checkpoint Inhibitor
IHC	ImmunoHistoChemistry
IO	Immuno-Oncology
IQR	InterQuartile Range
iTIL	Intratumoural Tumour-Infiltrating Lymphocyte
KM	Kaplan–Meier
KW	Kruskal–Wallis test
LCLC	Large Cell Lung Carcinoma
LRT	Likelihood Ratio Test
LUAD	LUng ADenocarcinoma
LUSC	LUng Squamous cell Carcinoma
MAF	Minor Allele Frequency
MAPK	Mitogen-Activated Protein Kinase
MNV	Multi-Nucleotide Variant
mOS	Median Overall Survival
NA	Not available
NE	Not evaluated
NGS	Next-Generation Sequencing
NSCLC	Non-Small Cell Lung Cancer
NSQ	Non-SQuamous NSCLC
OR	Odd Ratio
OS	Overall Survival
PCR	Polymerase Chain Reaction
PD-L1	Programmed Death-Ligand 1
PD1	Programmed Death-1/Programmed Cell Death Protein 1
PI3K	Phosphatidylinositol-4,5-Bisphosphate 3-Kinase
PIK3CA	Phosphatidylinositol-4,5-Bisphosphate 3-Kinase Catalytic Subunit Alpha
PV	Pathogenic Variant
Q1	Quartile 1
Q3	Quartile 3
RAF	Rapidly Accelerated in Sarcoma
RAS	Rat Sarcoma gene homologue
RB1	Retinoblastoma 1 Protein
rs	Spearman’s rank correlation coefficient
RTK	Receptor Tyrosine Kinase
SACT	Systemic Anti-Cancer Treatment
SCLC	Small Cell Lung Cancer
SCNA	Somatic Copy Number Alteration
SD	Standard Deviation
SNV	Single Nucleotide Variant
sTIL	stromal Tumour-Infiltrating Lymphocyte
TACS	TArget Capture Sequences
TCC	Tumour Cell Content
TGF-β	Transforming Growth Factor-beta
TIL	Tumour-Infiltrating Lymphocyte
TKI	Tyrosine Kinase Inhibitor
TNM	Tumour, Nodes, Metastases
TOPMed	Trans-Omics for Precision Medicine
TPS	Tumour Proportion Score
VAF	Variant Allele Frequency
VEP	Variant Effect Predictor
WHO	World Health Organisation
WNT	Wingless-related integration site
χ2	Pearson’s chi-squared test
Gene Name Abbreviations:	
*AKT1*	AKT Serine/Threonine Kinase 1
*ALK*	Anaplastic Lymphoma Kinase
*APC*	Adenomatous Polyposis Coli
*AR*	Androgen Receptor
*ARAF*	A-RAF/A- Rapidly Accelerated Fibrosarcoma
*ATM*	Ataxia-Telangiectasia Mutated
*BARD1*	BRCA1 (BReat CAncer gene 1)- associated RING (Really Interesting New Gene) domain protein 1
*BRAF*	B-RAF/B- Rapidly Accelerated Fibrosarcoma
*BRCA1*	BReat CAncer gene 1
*BRCA2*	BReast CAncer gene 2
*BRIP1*	BRCA1 (BReat CAncer gene 1) Interacting Protein 1
*CDKN2A*	Cyclin-dependent kinase inhibitor 2A
*CHEK2*	Checkpoint Kinase 2
*chr*	Chromosome
*CTNNB1*	Catenin beta-1
*CUL3*	Cullin 3
*DDR2*	Discoidin Domain Receptor 2
*DICER1*	Dicer 1, ribonuclease III
*EGFR*	Epidermal Growth Factor Receptor
*ERBB2*	Erb-B2 (avian erythroblastosis oncogene homologue) Receptor Tyrosine Kinase 2
*ERBB3*	Erb-B3 (avian erythroblastosis oncogene homologue) Receptor Tyrosine Kinase 3
*ERBB4*	Erb-B4 (avian erythroblastosis oncogene homologue) Receptor Tyrosine Kinase 4
*ESR1*	Estrogen Receptor 1
*FBXW7*	F-Box and WD Repeat Domain Containing 7
*FGFR1*	Fibroblast Growth Factor Receptor 1
*FGFR2*	Fibroblast Growth Factor Receptor 2
*FGFR3*	Fibroblast Growth Factor Receptor 3
*JAK2*	Janus Kinase 2
*KEAP1*	Katch-like ECH (Erythroid Cell-derived Cap’n’collar Homologue)-associated Protein 1
*KIT*	KIT proto-oncogene, receptor tyrosine kinase
*KRAS*	Kirsten Rat sarcoma viral oncogene homologue
*LKB1*	Liver Kinase B1
*MAP2K1*	Mitogen-Activated Protein Kinase 1
*MET*	N-methyl-N′-nitro-N-nitrosoguanidine (MNNG) Human Osteosarcoma Epithelial- mesenchymal Transition transforming gene
*MLH1*	MutL Homologue 1
*MRE11A*	Meiotic Recombination 11 Homologue A
*MSH6*	MutS Homolog 6
*MTOR*	Mammalian Target Of Rapamycin
*NBN*	Nibrin
*NF1*	Neurofibromin 1
*NFE2L2*	Nuclear Factor, Erythroid 2-Like 2
*NRAS*	Neuroblastoma RAt Sarcoma viral oncogene homologue
*NTRK1*	Neurotrophic Receptor Tyrosine Kinase 1
*NTRK2*	Neurotrophic Receptor Tyrosine Kinase 2
*NTRK3*	Neurotrophic Receptor Tyrosine Kinase 3
*PDGFRA*	Platelet Derived Growth Factor Receptor Alpha
*PIK3CA*	Phosphatidylinositol-4,5-Bisphosphate 3-Kinase Catalytic Subunit Alpha
*POLE*	Polymerase (DNA directed), Epsilon, Catalytic Subunit
*PTEN*	Phosphatase and Tensin homologue deleted on chromosome 10
*RAF1*	C-RAF/C- Rapidly Accelerated in Fibrosarcoma/v-raf-1 murine leukaemia viral oncogene homolog 1
*RET*	Rearranged during Transfection
*ROS1*	ROS proto-oncogene 1, receptor tyrosine kinase
*SMAD4*	C. elegans SMA (small worm phenotype) and Drosophila MAD (“Mothers Against Decapentaplegic”)
*SOX2*	SOX2 (SRY-Box Transcription Factor 2)
*STK11*	Serine/Threonine Kinase 11
*TERT*	Telomerase Reverse Transcriptase
*TP53*	Tumour Protein p53
*TP63*	Tumour Protein p63
