# Supplementary material for: Integrated Molecular, Clinicopathological and Immune Profiling of Non-Small Cell Lung Cancer: The MONREPOS Hellenic Cooperative Oncology Group (HeCOG) Study (HE_2TR/13)
Source: Int J Mol Sci. 2026 Sep 29;27(19):8724. doi: 10.3390/ijms27198724 (PMC13665066; doi:10.3390/ijms27198724)
Supplement: Supplementary file 1 [file ijms-27-08724-s001.zip › Supplementary Figures S1-S4.pdf]

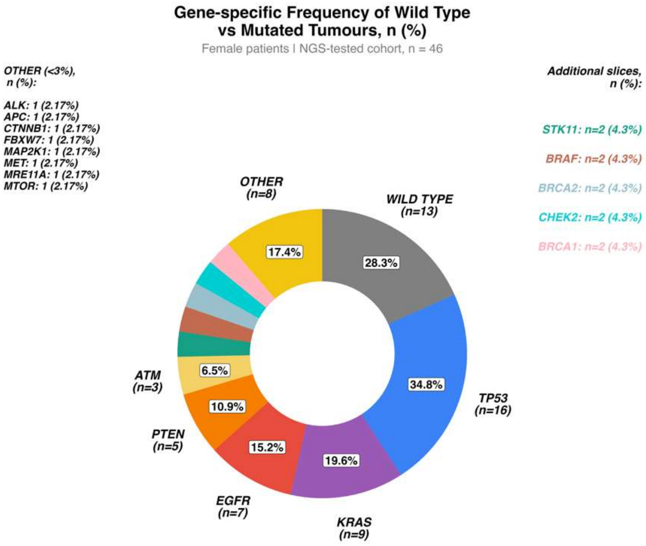

a. Frequency doughnut pie chart of mutated genes in female patients, %.

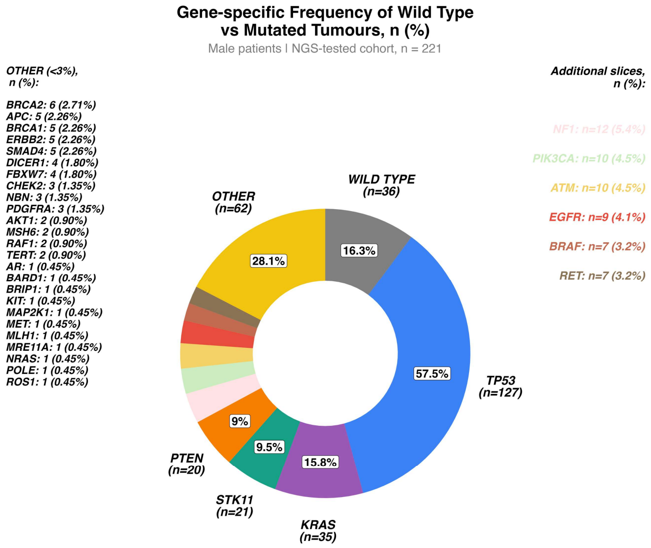

b. Frequency doughnut pie chart of mutated genes in male patients, %.

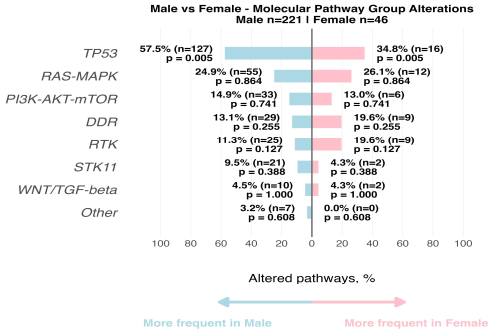

c. Pyramid bar chart with most frequently altered pathways in female vs male patients.

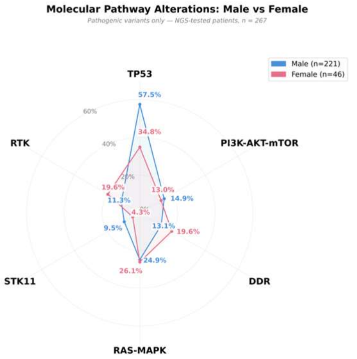

d. Radar plot with altered molecular pathways in female vs male patients, %.

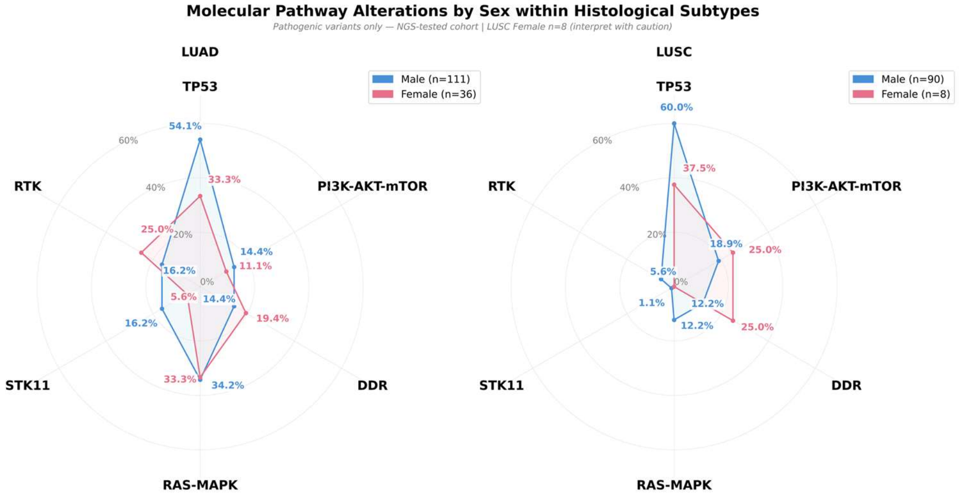

e. Radar plot with molecular pathways altered in LUAD (male/ female), and LUSC (male/ female), %.

Figure S1. Profiles of detected molecular alterations in male and female patients; a, b: doughnut pie charts, c: pyramid bar chart with statistical testing, d: radar plot, e: combined radar plots for LUAD and LUSC.

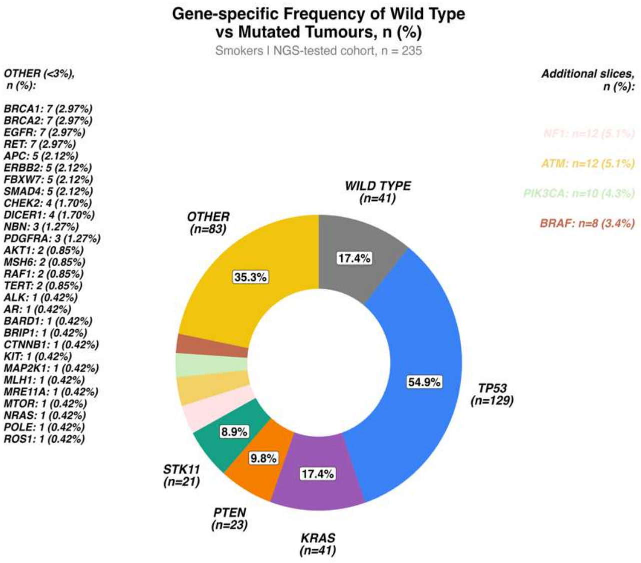

a. Frequency doughnut pie chart of mutated genes in smokers, %

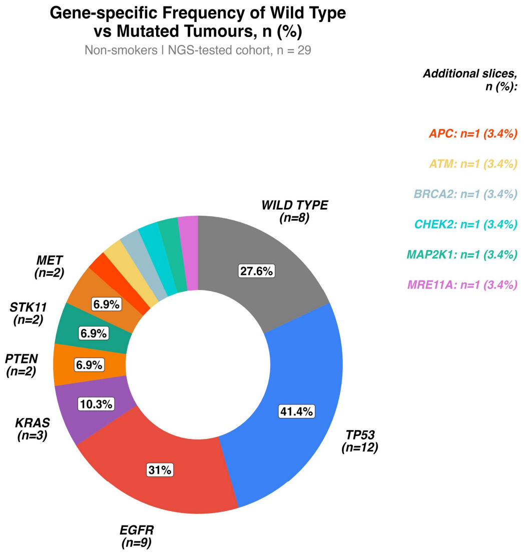

b. Frequency doughnut pie chart of mutated genes in non-smokers, %.

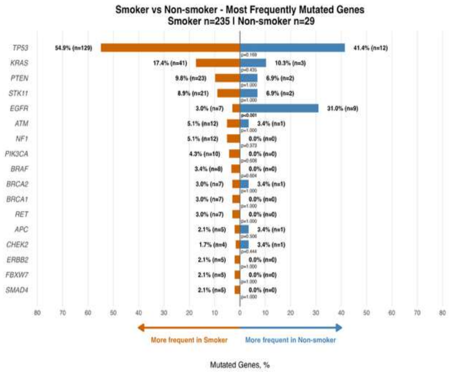

c. Pyramid bar chart with most frequently mutated genes in smokers vs non-smokers.

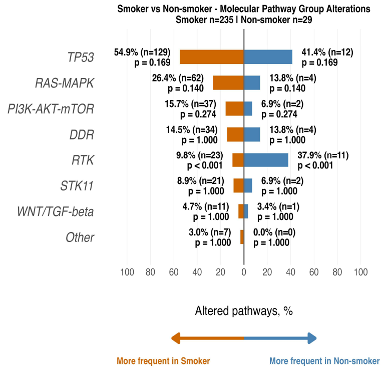

d. Pyramid bar chart with most frequently altered pathways in smokers vs non-smokers.

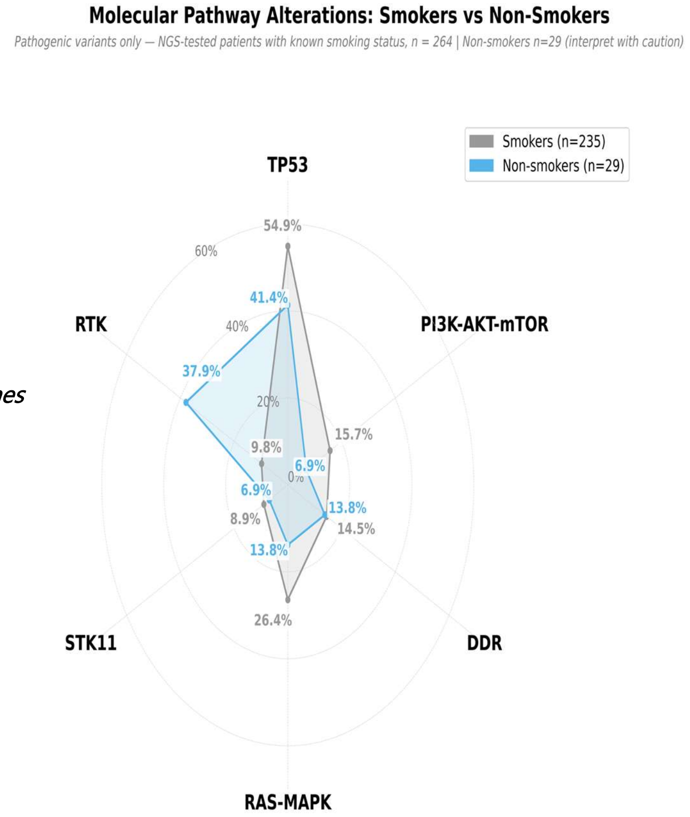

e. Radar plot with altered molecular pathways in smokers vs non-smokers, %.

Figure S2. Profiles of detected molecular alterations in smokers and non-smokers patients; a, b: doughnut pie charts, c, d: pyramid bar charts with statistical testing, e: combined radar plots smokers and non-smokers.

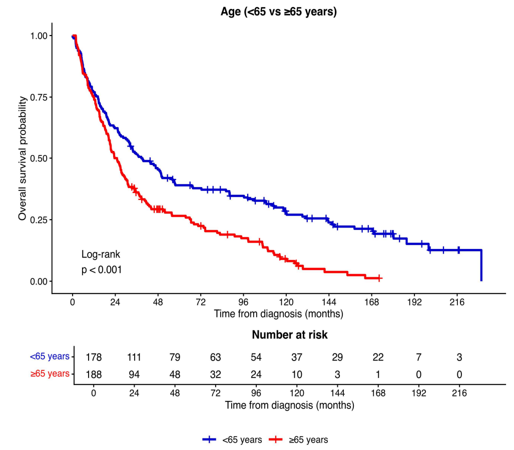

a. Age (65-year-old cut-off)

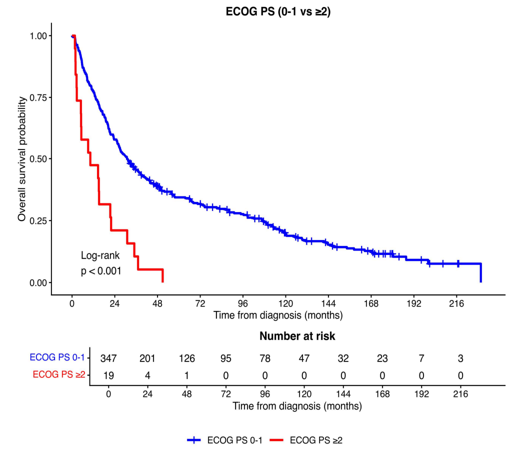

b. ECOG PS (0-1 vs ≥2)

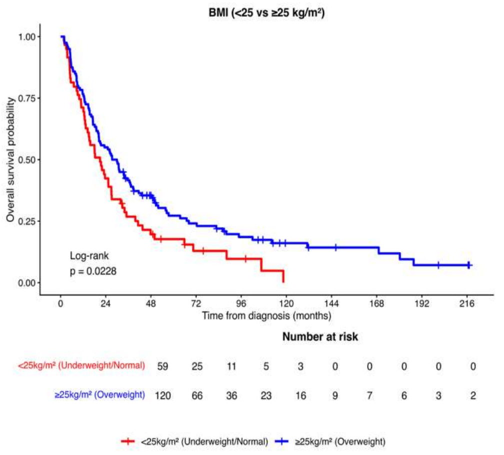

c. BMI (Underweight/ Normal VS Overweight)

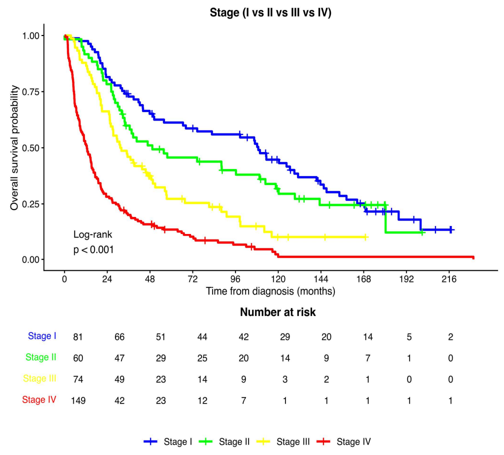

d. Stage (I, II, III, IV)

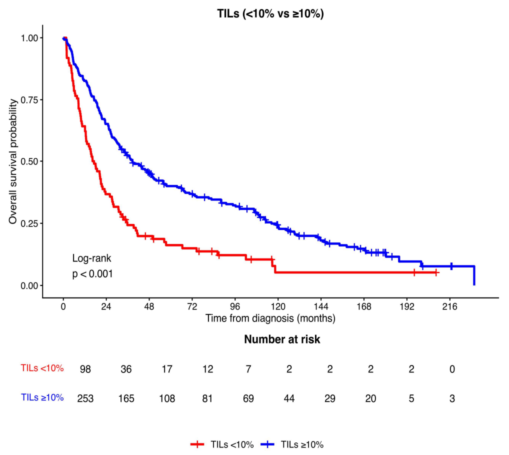

e. TILs (<10% VS ≥10%)

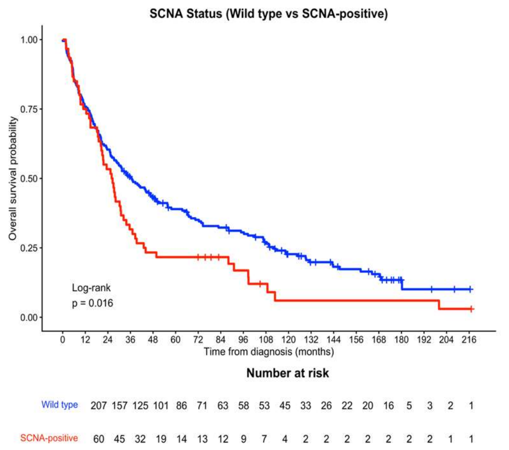

f. SCNA status (wild type vs SCNA-positive)

Figure S3. KM curves for key clinicopathological and biomarker variables; a. Age, b. ECOG PS, c. BMI, d. Stage, e. TILs, f. SCNA status.

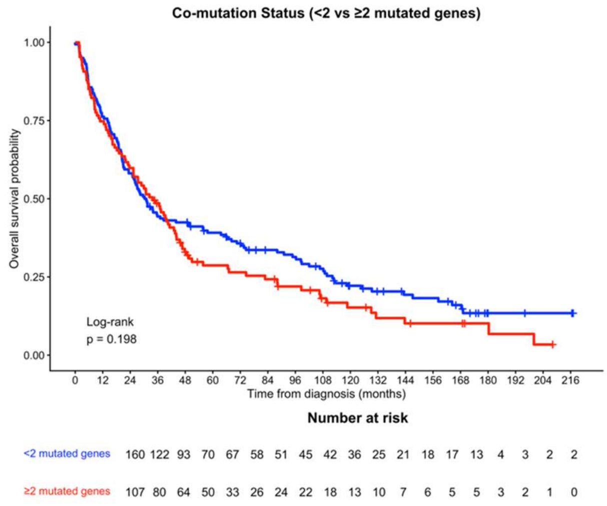

Figure S4. KM curves for Co-mutation status (<2 vs ≥2 mutated genes).
