# Supplementary material for: Integrated Molecular, Clinicopathological and Immune Profiling of Non-Small Cell Lung Cancer: The MONREPOS Hellenic Cooperative Oncology Group (HeCOG) Study (HE_2TR/13)
Source: Int J Mol Sci. 2026 Sep 29;27(19):8724. doi: 10.3390/ijms27198724 (PMC13665066; doi:10.3390/ijms27198724)
Supplement: Supplementary file 1 [file ijms-27-08724-s001.zip › Supplementary Tables S1-S16.pdf]

Table S1. Baseline clinicopathological characteristics, n=367.

**Patients characteristics**	**n (%)(1)**
**Age (years)**	
Mean (SD) | Median (range, IQR)	
64.4 (9.23) | 65 (35-109, 59-70)	
**Age group (years)**	
ASCO older individual cut-off	
<65 years old	178 (48.5%)
≥65 years old	189 (51.5%)
**Sex**	
Female	65 (17.7%)
Male	302 (82.3%)
**Smoking status**	
Non-smoker	35 (9.7%)
Smoker	325 (90.3%)
NA(2)	7 (1.9%)
**BMI baseline (kg/m^2)**	
Mean (SD) | Median (range, IQR)	
27.3 (5.0) | 26.8 (13.4-40.1, 23.9-29.8)	
**BMI baseline category (kg/m^2)**	
<25 (≤normal)	60 (32.8%)
≥25 (≥obese)	124 (67.4%)
NA	183 (49.9%)
**ECOG PS**	
0-1	348 (94.8%)
→ *0*	285 (77.7%)
→ *1*	63 (17.2%)
≥2	19 (5.2%)
→ *2*	16 (4.4%)
→ *3*	3 (0.8%)
**Tumour characteristics**	**n (%)**
**Histology**	
NSQ	238 (64.9%)
LUAD	*207 (56.4%)*
Other	*31 (8.4%)*
→*LCLC*	*17 (4.6%)*
→*Mixed*	*9 (2.5%)*
→*Unclassified*	*1 (0.3%)*
→*Undifferentiated*	*4 (1.1%)*
LUSC	129 (35.1%)

**Tumour characteristics (cont'd)**	**n (%)(1)**
**Grade**	
G1-2	124 (40.9%)
→ *G1*	*17 (5.6%)*
→ *G2*	*107 (35.3%)*
G3-4	179 (59.1%)
→ *G3*	*177 (58.4%)*
→ *G4*	*2 (0.7%)*
NA	64 (17.4%)
**Stage(3)**	
**TNM Stage**	
I-III (Non-metastatic)	216 (59.2%)
I	*82 (22.5%)*
II	*60 (16.4%)*
III	*74 (20.3%)*
→ *IIIa*	*45 (12.3%)*
→ *IIIb*	*26 (7.1%)*
→ *IIIc*	*3 (0.8%)*
IV (Metastatic)	149 (40.8%)
NA	2 (0.5%)
**T stage**	
T1-T2	163 (62.9%)
→ *T1*	*69 (26.6%)*
→ *T2*	*94 (36.3%)*
T3-T4	96 (37.1%)
→ *T3*	*59 (22.8%)*
→ *T4*	*37 (14.3%)*
NA	108 (29.4%)
**N stage**	
N negative	125 (47.9%)
N positive	136 (52.1%)
→ *N1*	*61 (23.4%)*
→ *N2*	*58 (22.2%)*
→ *N3*	*17 (6.5%)*
NA	106 (28.9%)
**M stage**	
M0	216 (59.2%)
M1	149 (40.8%)
NA	3 (0.8%)

(1) Percentages (%) for recorded cases use valid denominators. NA percentages (%) use total denominators, n=367. Valid percentages may not sum to exactly 100% due to one decimal rounding.
(2) NA corresponds to not available/ missing data.
(3) Stage refers to either clinical stage or pathological stage at diagnosis as per AJCC 8th edition.

Table S2. Inherent characteristics (sex, histology, grade), and disease primary causal factor (smoking status) crosstabulations, n=367.

Sex as inherent patient characteristic and smoking status as primary causal factor of disease

		**Sex**			**Smoking status**			
**Variable**	**All**, n (%)(1)	**Male**, n (%)(1)	**Female**, n (%)(1)	***p* value**(2)	**Non-smoker**, n (%)(1)	**Smoker**, n (%)(1)	**NA**(3), n (%)(1)	***p* value**(2)
**Age**				0.134$^{\chi^2}$				0.501$^{\chi^2}$
<65 years	178 (48.5%)	141 (46.7%)	37 (56.9%)		19 (54.3%)	157 (48.3%)	2 (28.6%)	
≥65 years	189 (51.5%)	161 (53.3%)	28 (43.1%)		16 (45.7%)	168 (51.7%)	5 (71.4%)	
**Smoking status**				**<0.001**$^{\chi^2}$				
Non-smoker	35 (9.7%)	12 (4.1%)	23 (35.9%)					
Smoker	325 (90.3%)	284 (95.9%)	41 (64.1%)					
NA(3)	7 (1.9%)	6 (2%)	1 (1.5%)					
**ECOG PS**				0.351^{FET}				0.401^{FET}
0-1	348 (94.8%)	288 (95.4%)	60 (92.3%)		32 (91.4%)	310 (95.4%)	6 (85.7%)	
≥2	19 (5.2%)	14 (4.6%)	5 (7.7%)		3 (8.6%)	15 (4.6%)	1 (14.3%)	
**Histology**				**0.005**$^{\chi^2}$				0.201$^{\chi^2}$
NSQ	238 (64.9%)	186 (61.6%)	52 (80.0%)		26 (74.3%)	206 (63.4%)	6 (85.7%)	
LUSC	129 (35.1%)	116 (38.4%)	13 (20.0%)		9 (25.7%)	119 (36.6%)	1 (14.3%)	
**Grade**				0.692$^{\chi^2}$				**0.036**$^{\chi^2}$
G1-2	124 (40.9%)	104 (41.4%)	20 (38.5%)		16 (59.3%)	104 (38.5%)	4 (66.7%)	
G3-4	179 (59.1%)	147 (58.6%)	32 (61.5%)		11 (40.7%)	166 (61.5%)	2 (33.3%)	
NA(3)	64 (17.4%)	51 (16.9%)	13 (20.0%)		8 (22.9%)	55 (16.9%)	1 (14.3%)	
**Stage**(4)				0.972$^{\chi^2}$				0.665$^{\chi^2}$
I-III	216 (59.2%)	178 (59.1%)	38 (59.4%)		22 (62.9%)	192 (59.1%)	2 (40%)	
IV	149 (40.8%)	123 (40.9%)	26 (40.6%)		13 (37.1%)	133 (40.9%)	3 (60%)	
NA(3)	2 (0.5%)	1 (0.3%)	1 (1.5%)		0 (0%)	0 (0%)	2 (28.6%)	

Inherent tumour characteristics

		**Histology**			**Grade**			
**Variable**	**All**, n (%)(1)	**NSQ**, n (%)(1)	**LUSC**, n (%)(1)	***p* value**(2)	**G1-2**, n (%)(1)	**G3-4**, n (%)(1)	**NA**(3), n (%)(1)	***p* value**(2)
**Age**				0.061$^{\chi^2}$				0.381$^{\chi^2}$
<65 years	178 (48.5%)	124 (52.1%)	54 (41.9%)		56 (45.2%)	90 (50.3%)	32 (50%)	
≥65 years	189 (51.5%)	114 (47.9%)	75 (58.1%)		68 (54.8%)	89 (49.7%)	32 (50%)	
**ECOG PS**				0.186$^{\chi^2}$				0.775$^{\chi^2}$
0-1	348 (94.8%)	223 (93.7%)	125 (96.9%)		118 (95.2%)	169 (94.4%)	61 (95.3%)	
≥2	19 (5.2%)	15 (6.3%)	4 (3.1%)		6 (4.8%)	10 (5.6%)	3 (4.7%)	
**Grade**				**0.003**$^{\chi^2}$				
G1-2	124 (40.9%)	65 (34.4%)	59 (51.8%)					
G3-4	179 (59.1%)	124 (65.6%)	55 (48.2%)					
NA(3)	64 (17.4%)	49 (20.6%)	15 (11.6%)					
**Stage**				0.054$^{\chi^2}$				**0.010**$^{\chi^2}$
I-III	216 (59.2%)	131 (55.5%)	85 (65.9%)		88 (72.1%)	103 (57.5%)	25 (39.1%)	
IV	149 (40.8%)	105 (44.5%)	44 (34.1%)		34 (27.9%)	76 (42.5%)	39 (60.9%)	
NA(3)	2 (0.5%)	2 (0.8%)	0 (0%)		2 (1.6%)	0 (0%)	0 (0%)	

Statistics for sex and smoking status crosstabulations

**Variable**	**Sex (Male/ Female)**				**Smoking status (Non-smoker/ Smoker)**			
	**n**	***p* value**	**Test & value**	**OR (95% CI)**	**n**	***p* value**	**Test & value**	**OR (95% CI)**
**Age (<65/ ≥65 years)**	367	0.134	χ^2; χ2(1)=2.243	0.66 (0.37-1.18)	360	0.501	χ^2; χ^2(1)=0.452	1.27 (0.59-2.74)
**Smoking status (non-smoker/ smoker)**	360	**<0.001**	χ^2; **χ^2(1)=60.947**	**0.08 (0.03-0.17)**				
**ECOG PS (0-1/ ≥2)**	367	0.351	FET; (-)	1.71 (0.46-5.27)	360	0.401	FET; (-)	0.52 (0.14-2.93)
**Histology (NSQ/ LUSC)**	367	**0.005**	χ^2; **χ^2(1)=7.953**	**0.40 (0.19-0.79)**	360	0.201	χ^2; χ^2(1)=1.639	1.67 (0.73-4.18)
**Grade (1-2/ 3-4)**	303	0.692	χ^2; χ^2(1)=0.157	1.13 (0.59-2.21)	297	**0.036**	**χ^2; χ^2(1)=4.385**	**2.31 (0.97-5.75)**
**Stage (I-III/ IV)**	365	0.972	χ^2; χ^2(1)=0.001	0.99 (0.55-1.77)	360	0.665	χ^2; χ^2(1)=0.187	1.17 (0.54-2.63)

Statistics for histology and grade crosstabulations

**Variable**	**Histology (NSQ/ LUSC)**				**Grade (1-2/ 3-4)**			
	**n**	***p* value**	**Test & value**	**OR (95% CI)**	**n**	***p* value**	**Test & value**	**OR (95% CI)**
**Age (<65/ ≥65 years)**	367	0.061	χ^2; χ^2(1)=3.512	1.51 (0.96-2.39)	303	0.381	χ^2; χ^2(1)=0.769	0.81 (0.50-1.32)
**ECOG PS (0-1/ ≥2)**	367	0.186	χ^2; χ^2(1)=1.747	0.48 (0.11-1.54)	303	0.775	χ^2; χ^2(1)=0.082	1.16 (0.37-4.01)
**Grade (1-2/ 3-4)**	303	**0.003**	χ^2; **χ^2(1)=8.867**	**0.49 (0.30-0.81)**				
**Stage (I-III/ IV)**	365	0.054	χ^2; χ^2(1)=3.722	0.65 (0.40-1.03)	301	**0.010**	χ^2; **χ^2(1)=6.659**	**1.91 (1.13-3.24)**

(1) Percentages (%) for recorded cases use valid denominators and are calculated within columns. NA percentages (%) use total denominators, n=367. Valid percentages may not sum to exactly 100% due to one decimal rounding.
(2) Statistical testing performed excluding NA. FET: Fisher's exact test, χ^2: Pearson's Chi-squared test.
(3) NA corresponds to not available/ missing data.
(4) Stage refers to either clinical or pathological stage at diagnosis.

Table S3. Comparison of patients with and without available next-generation sequencing, n=367[1].

Characteristic[2]	Not sequenced (n=100)	Sequenced (n=267)	*p* value	Test[3]
**Age**			0.411	χ^2
<65 years	45 (45.0)	133 (49.8)		
>=65 years	55 (55.0)	134 (50.2)		
**Sex**			0.692	χ^2
Female	19 (19.0)	46 (17.2)		
Male	81 (81.0)	221 (82.8)		
**Smoking status**			0.158	χ^2
Non-smoker	6 (6.2)	29 (11.0)		
Smoker	90 (93.8)	235 (89.0)		
NA	4	3		
**ECOG PS**			0.663	χ^2
0-1	94 (94.0)	254 (95.1)		
>=2	6 (6.0)	13 (4.9)		
**Histology**			0.308	χ^2
NSQ	69 (69.0)	169 (63.3)		
LUSC	31 (31.0)	98 (36.7)		
**Histologic grade**			0.608	χ^2
G1-2	30 (38.5)	94 (41.8)		
G3-4	48 (61.5)	131 (58.2)		
NA	22	42		
**Disease stage**			0.536	χ^2
I-III (non-metastatic)	56 (56.6)	160 (60.2)		
IV (metastatic)	43 (43.4)	106 (39.8)		
NA	1	1		
**T stage**			0.569	χ^2
T1-T2	42 (64.6)	121 (62.4)		
T3-T4	23 (35.4)	73 (37.6)		
NA	35	73		
**N stage**			0.046	χ^2
N negative	26 (38.8)	99 (51.0)		
N positive	41 (61.2)	95 (49.0)		
NA	33	73		
**Surgical resection**			0.117	χ^2
No	48 (48.0)	104 (39.0)		
Yes	52 (52.0)	163 (61.0)		
**Overall survival**			0.172	log-rank
Median OS, months (95% CI)	23.4 (18.8–35.0)	31.3 (26.5–39.3)		

[1] Data are n (%) unless stated. Percentages are calculated using valid denominators; NA rows give the number of patients with missing data.
[2] NA, not available; NSQ, non-squamous; LUSC, lung squamous cell carcinoma; ECOG PS, Eastern Cooperative Oncology Group performance status.
[3] χ^2: Pearson's chi-square test without continuity correction; log-rank: log-rank test.

Table S4. Biomarker distribution, n=367.

Optical microscopy biomarkers			
**PD-L1 expression**		**TIL expression**	
**PD-L1 cohort**	**Patients, n (%)[1]**	**TILs cohort**	**Patients, n (%)[1]**
Evaluated for PD-L1	298 (81.2%)	Evaluated for TILs	352 (95.9%)
NE[2]	69 (18.8%)	NE[2]	15 (4.1%)
**TPS (<1%, 1-50%, ≥50%)**		**TILs (%) 4 groups**	
<1%	235 (78.9%)	<10%	98 (27.8%)
1-50%	46 (15.4%)	10-25%	83 (23.6%)
≥50%	17 (5.7%)	25-50%	38 (10.8%)
**TPS (negative/ positive)**		≥50%	133 (37.8%)
Negative (<1%)	235 (78.9%)	**TILs (%) 10% cut-off**	
Positive (≥1%)	63 (21.1%)	<10%	98 (27.8%)
**TPS (<50%, ≥50%)**		≥10%	254 (72.2%)
<50%	281 (94.3%)	**TILs (%) 50% cut-off**	
≥50%	17 (5.7%)	<50%	219 (62.2%)
		≥50%	133 (37.8%)

Molecular biomarkers						
**Somatic PV/ likely PV detection**			**SCNA detection**			
**NGS cohort**		**Patients, n (%)[1]**	**NGS cohort**			**Patients, n (%)[1]**
NGS tested		n=267 (72.8%)	NGS tested			n=267 (72.8%)
NE[2]		n=100 (27.2%)	NE[2]			n=100 (27.2%)
**Gene PV status**			**SCNA status**			
Wild type		49 (18.4%)	No SCNA			207 (77.5%)
Mutated		218 (81.6%)	SCNA-positive			60 (22.5%)
→1 gene		111 (41.6%)	→1 SCNA			49 (18.4%)
→2 genes		72 (27%)	→ ≥2 SCNAs			11 (4.1%)
→3 genes		22 (8.2%)	→2 SCNAs			8 (3%)
→≥4 genes[3]		13 (4.9%)	→3 SCNAs			3 (1.1%)
**Most frequently detected genes with PVs**			**Most frequently detected SCNAs[4]**			
**Gene**	**Status**	**Patients, n (%)[1]**	**Cytoband**	**Gene**	**Status**	**Patients, n (%)[1]**
TP53	Mutated	143 (53.6%)	8p11.23	*FGFR1*	Gain	13 (4.9%)
	Wild type	124 (46.4%)			No SCNA	254 (95.1%)
KRAS	Mutated	44 (16.5%)	8q24.21	*MYC*	Gain	13 (4.9%)
	Wild type	223 (83.5%)			No SCNA	254 (95.1%)
PTEN	Mutated	25 (9.4%)	12p12.1	*KRAS*	Gain	11 (4.1%)
	Wild type	242 (90.6%)			No SCNA	256 (95.9%)
STK11	Mutated	23 (8.6%)	3q26.32	*PIK3CA*	Gain	11 (4.1%)
	Wild type	244 (91.4%)			No SCNA	256 (95.9%)
EGFR	Mutated	16 (6%)	17q12	*ERBB2*	Gain	7 (2.6%)
	Wild type	251 (94%)			No SCNA	260 (97.4%)
ATM	Mutated	13 (4.9%)	9p21.3	*CDKN2A*	Loss	6 (2.2%)
	Wild type	254 (95.1%)			No SCNA	261 (97.8%)
NF1	Mutated	12 (4.5%)	7p11.2	*EGFR*	Gain	6 (2.2%)
	Wild type	255 (95.5%)			No SCNA	261 (97.8%)
PIK3CA	Mutated	10 (3.7%)	4q12	*KIT*	Gain	4 (1.5%)
	Wild type	257 (96.3%)			No SCNA	263 (98.5%)

(1) Percentages (%) for recorded cases use valid denominators and are calculated within columns. NE percentages (%) use total denominators, n=367. Valid percentages may not sum to exactly 100% due to one decimal rounding.
(2) NE corresponds to patients whose tissue was not tested.
(3) PV in 4 genes: 11 patients (4.1%), PV in 5 genes: 1 patient (0.4%), PV in 8 genes: 1 patient (0.4%).
(4) Due to patients with ≥2 SCNAs detected, overall individual SCNA events across 60 patients equal 74.

Table S5. Individual gene PV detection within the LUAD/ LUSC[(1)], NGS[(2)] tested subset (Other histology excluded), n = 245.

Gene	Histology (LUAD vs LUSC)			*p* value[(4)]	Test value	OR (95% CI)
	LUAD/LUSC cohort (n = 245), n (%)[(3)]	LUAD (n = 147), n (%)[(3)]	LUSC (n = 98), n (%)[(3)]			
TP53	129 (52.7%)	72 (49.0%)	57 (58.2%)	0.158$^{\chi^2}$	$\chi^2(1)=1.989$	0.69 (0.40–1.19)
KRAS	41 (16.7%)	38 (25.9%)	3 (3.1%)	**<0.001**$^{\chi^2}$	$\chi^2(1)=21.915$	10.96 (3.31–57.27)
PTEN	25 (10.2%)	17 (11.6%)	8 (8.2%)	0.389$^{\chi^2}$	$\chi^2(1)=0.742$	1.47 (0.57–4.11)
STK11	21 (8.6%)	20 (13.6%)	1 (1.0%)	**<0.001**$^{\chi^2}$	$\chi^2(1)=11.884$	15.17 (2.34–637.71)
EGFR	16 (6.5%)	15 (10.2%)	1 (1.0%)	**0.004**$^{\chi^2}$	$\chi^2(1)=8.124$	10.96 (1.63–467.75)
ATM	13 (5.3%)	9 (6.1%)	4 (4.1%)	0.485$^{\chi^2}$	$\chi^2(1)=0.487$	1.53 (0.41–7.00)
NF1	10 (4.1%)	7 (4.8%)	3 (3.1%)	0.744^{FET}	-	1.58 (0.35–9.71)
PIK3CA	10 (4.1%)	4 (2.7%)	6 (6.1%)	0.205^{FET}	-	0.43 (0.09–1.87)
BRAF	8 (3.3%)	7 (4.8%)	1 (1.0%)	0.150^{FET}	-	4.83 (0.60–220.58)
BRCA1	7 (2.9%)	3 (2.0%)	4 (4.1%)	0.442^{FET}	-	0.49 (0.07–2.97)
BRCA2	7 (2.9%)	4 (2.7%)	3 (3.1%)	1.000^{FET}	-	0.89 (0.15–6.19)
RET	7 (2.9%)	5 (3.4%)	2 (2.0%)	0.705^{FET}	-	1.69 (0.27–18.05)
APC	6 (2.4%)	3 (2.0%)	3 (3.1%)	0.686^{FET}	-	0.66 (0.09–5.04)
CHEK2	5 (2.0%)	5 (3.4%)	0 (0.0%)	0.160^{FET}	-	-
FBXW7	5 (2.0%)	0 (0.0%)	5 (5.1%)	**0.010**^{FET}	-	0.00 (0.00–0.71)
SMAD4	4 (1.6%)	3 (2.0%)	1 (1.0%)	0.652^{FET}	-	2.02 (0.16–107.11)
DICER1	3 (1.2%)	2 (1.4%)	1 (1.0%)	1.000^{FET}	-	1.34 (0.07–79.70)
ERBB2	3 (1.2%)	3 (2.0%)	0 (0.0%)	0.277^{FET}	-	-
PDGFRA	3 (1.2%)	3 (2.0%)	0 (0.0%)	0.277^{FET}	-	-
AKT1	2 (0.8%)	0 (0.0%)	2 (2.0%)	0.159^{FET}	-	0.00 (0.00–3.54)
MAP2K1	2 (0.8%)	1 (0.7%)	1 (1.0%)	1.000^{FET}	-	0.67 (0.01–52.66)
MET	2 (0.8%)	1 (0.7%)	1 (1.0%)	1.000^{FET}	-	0.67 (0.01–52.66)
MRE11A	2 (0.8%)	2 (1.4%)	0 (0.0%)	0.518^{FET}	-	-
MSH6	2 (0.8%)	0 (0.0%)	2 (2.0%)	0.159^{FET}	-	0.00 (0.00–3.54)
NBN	2 (0.8%)	2 (1.4%)	0 (0.0%)	0.518^{FET}	-	-
RAF1	2 (0.8%)	0 (0.0%)	2 (2.0%)	0.159^{FET}	-	0.00 (0.00–3.54)
TERT	2 (0.8%)	1 (0.7%)	1 (1.0%)	1.000^{FET}	-	0.67 (0.01–52.66)
ALK	1 (0.4%)	1 (0.7%)	0 (0.0%)	1.000^{FET}	-	-
AR	1 (0.4%)	1 (0.7%)	0 (0.0%)	1.000^{FET}	-	-
BARD1	1 (0.4%)	0 (0.0%)	1 (1.0%)	0.400^{FET}	-	0.00 (0.00–26.00)
BRIP1	1 (0.4%)	0 (0.0%)	1 (1.0%)	0.400^{FET}	-	0.00 (0.00–26.00)
CTNNB1	1 (0.4%)	1 (0.7%)	0 (0.0%)	1.000^{FET}	-	-
KIT	1 (0.4%)	0 (0.0%)	1 (1.0%)	0.400^{FET}	-	0.00 (0.00–26.00)
MLH1	1 (0.4%)	1 (0.7%)	0 (0.0%)	1.000^{FET}	-	-
MTOR	1 (0.4%)	1 (0.7%)	0 (0.0%)	1.000^{FET}	-	-
NRAS	1 (0.4%)	0 (0.0%)	1 (1.0%)	0.400^{FET}	-	0.00 (0.00–26.00)
POLE	1 (0.4%)	1 (0.7%)	0 (0.0%)	1.000^{FET}	-	-
ROS1	1 (0.4%)	1 (0.7%)	0 (0.0%)	1.000^{FET}	-	-

(1) n = 336 tumours with LUAD or LUSC histology.
(2) NGS performed for 267 patients, n = 245 patients with NGS tested tumours and LUAD or LUSC histology.
(3) Percentages (%) for recorded cases are calculated within columns. Gene PVs ordered by descending detection frequency.
(4) Statistical testing performed within the LUAD/LUSC cohort. FET: Fisher's exact test, χ2: Pearson's Chi-squared test.

Table S6. Gene PV Status Distribution (wild type/ mutated) by Clinical and Tumour Characteristics, n=367.

		Gene PV status					
	**All,** n (%)[(1)]	**Wild type,** n (%)[(1)]	**Mutated,** n (%)[1)]	**NE**[(4)], n (%)[1)]	***p* value**	**Test value**[(2)]	**OR (95% CI)**
**CLINICAL CHARACTERISTICS**							
**Sex**					0.056^{χ^2}	$\chi^2(1)=3.642$	0.50 (0.23-1.13)
Male	302 (82.3%)	36 (73.5%)	185 (84.9%)	81 (81%)			
Female	65 (17.7%)	13 (26.5%)	33 (15.1%)	19 (19%)			
**Age**					0.413^{χ^2}	$\chi^2(1)=0.672$	1.30 (0.66-2.55)
<65 years	178 (48.5%)	27 (55.1%)	106 (48.6%)	45 (45%)			
≥65 years	189 (51.5%)	22 (44.9%)	112 (51.4%)	55 (55%)			
**Smoking status**					0.185^{χ^2}	$\chi^2(1)=1.756$	1.80 (0.64-4.60)
Non-smoker	35 (9.7%)	8 (16.3%)	21 (9.8%)	6 (6.2%)			
Smoker	325 (90.3%)	41 (83.7%)	194 (90.2%)	90 (93.8%)			
NA[(3)]	7 (1.9%)	0 (0%)	3 (1.4%)	4 (4%)			
**ECOG PS (0-1 vs ≥2)**					0.135^{FET}	-	-
0-1	348 (94.8%)	49 (100%)	205 (94%)	94 (94%)			
≥2	19 (5.2%)	0 (0%)	13 (6%)	6 (6%)			
**TUMOR CHARACTERISTICS**							
**Histology 3-tier (LUAD vs LUSC vs Other)**					0.252^{FET}	-	-
LUAD	207 (56.4%)	22 (44.9%)	125 (57.3%)	60 (60%)			
LUSC	129 (35.1%)	23 (46.9%)	75 (34.4%)	31 (31%)			
Other	31 (8.4%)	4 (8.2%)	18 (8.3%)	9 (9%)			
**Histology 2-tier a (NSQ vs LUSC)**					0.100^{χ^2}	$\chi^2(1)=2.706$	0.59 (0.30-1.17)
NSQ	238 (64.9%)	26 (53.1%)	143 (65.6%)	69 (69%)			
LUSC	129 (35.1%)	23 (46.9%)	75 (34.4%)	31 (31%)			
**Histology excluding Other (LUAD vs LUSC)**					0.092^{χ^2}	$\chi^2(1)=2.836$	0.58 (0.28-1.16)
LUAD	207 (61.6%)	22 (48.9%)	125 (62.5%)	60 (65.9%)			
LUSC	129 (38.4%)	23 (51.1%)	75 (37.5%)	31 (34.1%)			
**Grade 2-tier (low: G1-2 vs high: G3-4)**					0.722^{χ^2}	$\chi^2(1)=0.127$	1.13 (0.54-2.32)
Low grade (G1-2)	124 (40.9%)	19 (44.2%)	75 (41.2%)	30 (38.5%)			
High grade (G3-4)	179 (59.1%)	24 (55.8%)	107 (58.8%)	48 (61.5%)			
NA[(3)]	64 (17.4%)	6 (12.2%)	36 (16.5%)	22 (22%)			
**Stage 2-tier (I-III vs IV)** [(5)]					0.424^{χ^2}	$\chi^2(1)=0.639$	0.78 (0.40-1.53)
I-III	216 (59.2%)	27 (55.1%)	133 (61.3%)	56 (56.6%)			
IV	149 (40.8%)	22 (44.9%)	84 (38.7%)	43 (43.4%)			
NA[(3)]	2 (0.5%)	0 (0%)	1 (0.5%)	1 (1%)			
**BIOMARKERS**							
**PD-L1 expression 2-tier a (<1% vs ≥1%)**					0.788^{χ^2}	$\chi^2(1)=0.072$	0.89 (0.38-2.30)
PDL1 negative (<1%)	235 (78.9%)	32 (78.0%)	163 (79.9%)	40 (75.5%)			
PDL1 positive (≥1%)	63 (21.1%)	9 (22.0%)	41 (20.1%)	13 (24.5%)			
NE[(4)]	69 (18.8%)	8 (16.3%)	14 (6.4%)	47 (47%)			
**PD-L1 expression 2-tier b (<50% vs ≥50%)**					0.081^{FET}	-	-
PDL1 <50%	281 (94.3%)	41 (100%)	188 (92.2%)	52 (98.1%)			
PDL1 ≥50%	17 (5.7%)	0 (0%)	16 (7.8%)	1 (1.9%)			
NE[(4)]	69 (18.8%)	8 (16.3%)	14 (6.4%)	47 (47%)			
**PD-L1 expression 3-tier (<1%/ 1-50%/ ≥50%)**							
PDL1 <1%	235 (78.9%)	32 (78%)	163 (79.9%)	40 (75.5%)	0.541^{FET}	-	-
PDL1 1-50%	46 (15.4%)	9 (22%)	25 (12.3%)	12 (22.6%)			
PDL1 ≥50%	17 (5.7%)	0 (0%)	16 (7.8%)	1 (1.9%)			
NE[(4)]	69 (18.8%)	8 (16.3%)	14 (6.4%)	47 (47%)			
**TIL expression 2-tier a (<10% vs ≥10%)**					0.327^{χ^2}	$\chi^2(1)=0.960$	0.69 (0.29-1.52)
TILs <10%	98 (27.8%)	10 (20.8%)	58 (27.8%)	30 (31.6%)			
TILs ≥10%	254 (72.2%)	38 (79.2%)	151 (72.2%)	65 (68.4%)			
NE[(4)]	15 (4.1%)	1 (2%)	9 (4.1%)	5 (5%)			
**TIL expression 2-tier b (<50% vs ≥50%)**					0.138^{χ^2}	$\chi^2(1)=2.200$	0.62 (0.31-1.23)
TILS <50%	219 (62.2%)	25 (52.1%)	133 (63.6%)	61 (64.2%)			
TILS ≥50%	133 (37.8%)	23 (47.9%)	76 (36.4%)	34 (35.8%)			
NE[(4)]	15 (4.1%)	1 (2%)	9 (4.1%)	5 (5%)			
**SCNA status (wild type vs SCNA-positive)**					0.708^{χ^2}	$\chi^2(1)=0.140$	0.87 (0.41-1.98)
Wild type	207 (77.5%)	37 (75.5%)	170 (78%)	0 (0%)			
SCNA-positive	60 (22.5%)	12 (24.5%)	48 (22%)	0 (0%)			
NE	100 (27.2%)	0 (0%)	0 (0%)	100 (100%)			

[(1)] Percentages (%) for recorded cases use valid denominators and are calculated within columns. NA percentages (%) use total denominators, n=367. Valid percentages may not sum to exactly 100% due to one decimal rounding.
[(2)] Statistical testing performed excluding NA & NE. FET: Fisher's exact test, χ^2: Pearson's Chi-squared test.
[(3)] NA corresponds to not available/ missing data.
[(4)] NE corresponds to patients whose tissue was not tested.
[(5)] Stage refers to either clinical or pathological stage at diagnosis.

Table S7. Co-mutation pairs detected within the NGS tested cohort. Distribution as per histology & sex, n = 267.

**Rank**(1)	**Overall (n = 267)**, n (%)(2)	**LUAD (n = 147)**, n (%)(2)	**LUSC (n = 98)**, n (%)(2)	**NSQ (n = 169)**, n (%)(2)	**Male (n = 221)**, n (%)(2)	**Female (n = 46)**, n (%)(2)
1	*PTEN + TP53*<br>15 (5.6%)	*KRAS + STK11*<br>12 (8.2%)	*PTEN + TP53*<br>6 (6.1%)	*KRAS + STK11*<br>13 (7.7%)	*PTEN + TP53*<br>14 (6.3%)	*EGFR + TP53*<br>4 (8.7%)
2	*KRAS + STK11*<br>13 (4.9%)	*KRAS + TP53*<br>10 (6.8%)	*PIK3CA + TP53*<br>5 (5.1%)	*KRAS + TP53*<br>11 (6.5%)	*KRAS + STK11*<br>12 (5.4%)	*ATM + KRAS*<br>2 (4.3%)
3	*KRAS + TP53*<br>12 (4.5%)	*EGFR + TP53*<br>9 (6.1%)	*FBXW7 + TP53*<br>4 (4.1%)	*EGFR + TP53*<br>9 (5.3%)	*KRAS + TP53*<br>10 (4.5%)	*BRCA2 + TP53*<br>2 (4.3%)
4	*EGFR + TP53*<br>10 (3.7%)	*PTEN + TP53*<br>9 (6.1%)	*APC + TP53*<br>3 (3.1%)	*PTEN + TP53*<br>9 (5.3%)	*PIK3CA + TP53*<br>7 (3.2%)	*KRAS + TP53*<br>2 (4.3%)
5	*PIK3CA + TP53*<br>7 (2.6%)	*KRAS + PTEN*<br>6 (4.1%)	*AKT1 + TP53*<br>2 (2.0%)	*KRAS + PTEN*<br>6 (3.6%)	*EGFR + TP53*<br>6 (2.7%)	*APC + MRE11A*<br>1 (2.2%)
6	*KRAS + PTEN*<br>6 (2.2%)	*ATM + KRAS*<br>5 (3.4%)	*ATM + TP53*<br>2 (2.0%)	*ATM + KRAS*<br>5 (3.0%)	*NF1 + TP53*<br>6 (2.7%)	*ATM + BRCA2*<br>1 (2.2%)
7	*NF1 + TP53*<br>6 (2.2%)	*NF1 + PTEN*<br>5 (3.4%)	*BRCA1 + TP53*<br>2 (2.0%)	*NF1 + PTEN*<br>5 (3.0%)	*KRAS + PTEN*<br>5 (2.3%)	*ATM + CTNNB1*<br>1 (2.2%)
8	*ATM + KRAS*<br>5 (1.9%)	*ATM + PTEN*<br>4 (2.7%)	*BRCA2 + TP53*<br>2 (2.0%)	*NF1 + TP53*<br>5 (3.0%)	*NF1 + PTEN*<br>5 (2.3%)	*ATM + FBXW7*<br>1 (2.2%)
9	*BRCA1 + TP53*<br>5 (1.9%)	*NF1 + TP53*<br>4 (2.7%)	*MSH6 + TP53*<br>2 (2.0%)	*ATM + PTEN*<br>4 (2.4%)	*APC + TP53*<br>4 (1.8%)	*ATM + PTEN*<br>1 (2.2%)
10	*NF1 + PTEN*<br>5 (1.9%)	*PTEN + RET*<br>4 (2.7%)	*APC + ATM*<br>1 (1.0%)	*ERBB2 + TP53*<br>4 (2.4%)	*BRCA1 + TP53*<br>4 (1.8%)	*ATM + TP53*<br>1 (2.2%)
11	*APC + TP53*<br>4 (1.5%)	*RET + TP53*<br>4 (2.7%)	*APC + KRAS*<br>1 (1.0%)	*PTEN + RET*<br>4 (2.4%)	*DICER1 + TP53*<br>4 (1.8%)	*BRAF + MTOR*<br>1 (2.2%)
12	*ATM + PTEN*<br>4 (1.5%)	*ATM + STK11*<br>3 (2.0%)	*ATM + BRCA2*<br>1 (1.0%)	*RET + TP53*<br>4 (2.4%)	*ERBB2 + TP53*<br>4 (1.8%)	*BRAF + PTEN*<br>1 (2.2%)
13	*BRAF + TP53*<br>4 (1.5%)	*BRAF + TP53*<br>3 (2.0%)	*ATM + FBXW7*<br>1 (1.0%)	*ATM + STK11*<br>3 (1.8%)	*PTEN + RET*<br>4 (1.8%)	*BRAF + TP53*<br>1 (2.2%)
14	*DICER1 + TP53*<br>4 (1.5%)	*BRCA1 + TP53*<br>3 (2.0%)	*ATM + RAF1*<br>1 (1.0%)	*BRAF + TP53*<br>3 (1.8%)	*RET + TP53*<br>4 (1.8%)	*BRCA1 + TP53*<br>1 (2.2%)
15	*ERBB2 + TP53*<br>4 (1.5%)	*NF1 + RET*<br>3 (2.0%)	*BARD1 + BRCA1*<br>1 (1.0%)	*BRCA1 + TP53*<br>3 (1.8%)	*ATM + KRAS*<br>3 (1.4%)	*BRCA2 + EGFR*<br>1 (2.2%)
16	*FBXW7 + TP53*<br>4 (1.5%)	*ATM + BRCA2*<br>2 (1.4%)	*BARD1 + TP53*<br>1 (1.0%)	*DICER1 + TP53*<br>3 (1.8%)	*ATM + PTEN*<br>3 (1.4%)	*BRCA2 + FBXW7*<br>1 (2.2%)
17	*PTEN + RET*<br>4 (1.5%)	*ATM + CHEK2*<br>2 (1.4%)	*BRAF + TP53*<br>1 (1.0%)	*NF1 + RET*<br>3 (1.8%)	*ATM + STK11*<br>3 (1.4%)	*BRCA2 + MAP2K1*<br>1 (2.2%)
18	*RET + TP53*<br>4 (1.5%)	*ATM + NF1*<br>2 (1.4%)	*BRCA2 + EGFR*<br>1 (1.0%)	*STK11 + TP53*<br>3 (1.8%)	*BRAF + TP53*<br>3 (1.4%)	*CHEK2 + EGFR*<br>1 (2.2%)
19	*ATM + BRCA2*<br>3 (1.1%)	*BRAF + PTEN*<br>2 (1.4%)	*BRCA2 + FBXW7*<br>1 (1.0%)	*ATM + BRCA2*<br>2 (1.2%)	*FBXW7 + TP53*<br>3 (1.4%)	*CHEK2 + KRAS*<br>1 (2.2%)
20	*ATM + STK11*<br>3 (1.1%)	*BRAF + STK11*<br>2 (1.4%)	*BRIP1 + PIK3CA*<br>1 (1.0%)	*ATM + CHEK2*<br>2 (1.2%)	*NF1 + RET*<br>3 (1.4%)	*CTNNB1 + KRAS*<br>1 (2.2%)
21	*BRCA2 + TP53*<br>3 (1.1%)	*BRCA2 + PDGFRA*<br>2 (1.4%)	*BRIP1 + TP53*<br>1 (1.0%)	*ATM + NF1*<br>2 (1.2%)	*PTEN + STK11*<br>3 (1.4%)	*CTNNB1 + PTEN*<br>1 (2.2%)
22	*NF1 + RET*<br>3 (1.1%)	*CHEK2 + KRAS*<br>2 (1.4%)	*DICER1 + TP53*<br>1 (1.0%)	*BRAF + PTEN*<br>2 (1.2%)	*STK11 + TP53*<br>3 (1.4%)	*EGFR + MAP2K1*<br>1 (2.2%)
23	*PTEN + STK11*<br>3 (1.1%)	*CHEK2 + PTEN*<br>2 (1.4%)	*EGFR + TP53*<br>1 (1.0%)	*BRAF + STK11*<br>2 (1.2%)	*AKT1 + TP53*<br>2 (0.9%)	*EGFR + PTEN*<br>1 (2.2%)
24	*STK11 + TP53*<br>3 (1.1%)	*DICER1 + TP53*<br>2 (1.4%)	*FBXW7 + NF1*<br>1 (1.0%)	*BRCA2 + KRAS*<br>2 (1.2%)	*ATM + BRCA2*<br>2 (0.9%)	*FBXW7 + TP53*<br>1 (2.2%)
25	*AKT1 + TP53*<br>2 (0.7%)	*ERBB2 + TP53*<br>2 (1.4%)	*FBXW7 + PTEN*<br>1 (1.0%)	*BRCA2 + PDGFRA*<br>2 (1.2%)	*ATM + CHEK2*<br>2 (0.9%)	*KRAS + PTEN*<br>1 (2.2%)
26	*ATM + CHEK2*<br>2 (0.7%)	*KRAS + NF1*<br>2 (1.4%)	*KIT + TP53*<br>1 (1.0%)	*CHEK2 + KRAS*<br>2 (1.2%)	*ATM + NF1*<br>2 (0.9%)	*KRAS + STK11*<br>1 (2.2%)
27	*ATM + NF1*<br>2 (0.7%)	*PDGFRA + PTEN*<br>2 (1.4%)	*KRAS + TP53*<br>1 (1.0%)	*CHEK2 + PTEN*<br>2 (1.2%)	*BRAF + STK11*<br>2 (0.9%)	*MAP2K1 + TP53*<br>1 (2.2%)
28	*ATM + TP53*<br>2 (0.7%)	*PDGFRA + RET*<br>2 (1.4%)	*MSH6 + PIK3CA*<br>1 (1.0%)	*KRAS + NF1*<br>2 (1.2%)	*BRCA2 + KRAS*<br>2 (0.9%)	*MTOR + PTEN*<br>1 (2.2%)
29	*BRAF + PTEN*<br>2 (0.7%)	*PIK3CA + TP53*<br>2 (1.4%)	*MSH6 + TERT*<br>1 (1.0%)	*NBN + TP53*<br>2 (1.2%)	*BRCA2 + PDGFRA*<br>2 (0.9%)	*PTEN + TP53*<br>1 (2.2%)
30	*BRAF + STK11*<br>2 (0.7%)	*PTEN + STK11*<br>2 (1.4%)	*NF1 + TP53*<br>1 (1.0%)	*PDGFRA + PTEN*<br>2 (1.2%)	*CHEK2 + PTEN*<br>2 (0.9%)	
31	*BRCA2 + EGFR*<br>2 (0.7%)	*STK11 + TP53*<br>2 (1.4%)	*NRAS + TP53*<br>1 (1.0%)	*PDGFRA + RET*<br>2 (1.2%)	*KRAS + NF1*<br>2 (0.9%)	
32	*BRCA2 + KRAS*<br>2 (0.7%)	*APC + AR*<br>1 (0.7%)	*PIK3CA + PTEN*<br>1 (1.0%)	*PIK3CA + TP53*<br>2 (1.2%)	*MSH6 + TP53*<br>2 (0.9%)	
33	*BRCA2 + PDGFRA*<br>2 (0.7%)	*APC + MRE11A*<br>1 (0.7%)	*PIK3CA + STK11*<br>1 (1.0%)	*PTEN + STK11*<br>2 (1.2%)	*NBN + TP53*<br>2 (0.9%)	
34	*CHEK2 + KRAS*<br>2 (0.7%)	*APC + RET*<br>1 (0.7%)	*PIK3CA + TERT*<br>1 (1.0%)	*SMAD4 + TP53*<br>2 (1.2%)	*PDGFRA + PTEN*<br>2 (0.9%)	
35	*CHEK2 + PTEN*<br>2 (0.7%)	*APC + TP53*<br>1 (0.7%)	*PTEN + STK11*<br>1 (1.0%)	*APC + AR*<br>1 (0.6%)	*PDGFRA + RET*<br>2 (0.9%)	
36	*KRAS + NF1*<br>2 (0.7%)	*AR + RET*<br>1 (0.7%)	*TERT + TP53*<br>1 (1.0%)	*APC + MRE11A*<br>1 (0.6%)	*PIK3CA + PTEN*<br>2 (0.9%)	

Rank[1]	**Overall (n = 267)**, n (%)[2]	**LUAD (n = 147)**, n (%)[2]	**LUSC (n = 98)**, n (%)[2]	**NSQ (n = 169)**, n (%)[2]	**Male (n = 221)**, n (%)[2]	**Female (n = 46)**, n (%)[2]
37	*MSH6 + TP53*<br>*2 (0.7%)*	*AR + TP53*<br>*1 (0.7%)*		*APC + RET*<br>*1 (0.6%)*	*SMAD4 + TP53*<br>*2 (0.9%)*	
38	*NBN + TP53*<br>*2 (0.7%)*	*ATM + CTNNB1*<br>*1 (0.7%)*		*APC + TP53*<br>*1 (0.6%)*	*TERT + TP53*<br>*2 (0.9%)*	
39	*PDGFRA + PTEN*<br>*2 (0.7%)*	*ATM + EGFR*<br>*1 (0.7%)*		*AR + RET*<br>*1 (0.6%)*	*APC + AR*<br>*1 (0.5%)*	
40	*PDGFRA + RET*<br>*2 (0.7%)*	*ATM + PDGFRA*<br>*1 (0.7%)*		*AR + TP53*<br>*1 (0.6%)*	*APC + ATM*<br>*1 (0.5%)*	
41	*PIK3CA + PTEN*<br>*2 (0.7%)*	*ATM + PIK3CA*<br>*1 (0.7%)*		*ATM + CTNNB1*<br>*1 (0.6%)*	*APC + KRAS*<br>*1 (0.5%)*	
42	*SMAD4 + TP53*<br>*2 (0.7%)*	*ATM + RET*<br>*1 (0.7%)*		*ATM + EGFR*<br>*1 (0.6%)*	*APC + RET*<br>*1 (0.5%)*	
43	*TERT + TP53*<br>*2 (0.7%)*	*ATM + SMAD4*<br>*1 (0.7%)*		*ATM + PDGFRA*<br>*1 (0.6%)*	*AR + RET*<br>*1 (0.5%)*	
44	*APC + AR*<br>*1 (0.4%)*	*BRAF + KRAS*<br>*1 (0.7%)*		*ATM + PIK3CA*<br>*1 (0.6%)*	*AR + TP53*<br>*1 (0.5%)*	
45	*APC + ATM*<br>*1 (0.4%)*	*BRAF + MTOR*<br>*1 (0.7%)*		*ATM + RET*<br>*1 (0.6%)*	*ATM + EGFR*<br>*1 (0.5%)*	
46	*APC + KRAS*<br>*1 (0.4%)*	*BRCA1 + NF1*<br>*1 (0.7%)*		*ATM + SMAD4*<br>*1 (0.6%)*	*ATM + PDGFRA*<br>*1 (0.5%)*	
47	*APC + MRE11A*<br>*1 (0.4%)*	*BRCA1 + PTEN*<br>*1 (0.7%)*		*BRAF + KRAS*<br>*1 (0.6%)*	*ATM + PIK3CA*<br>*1 (0.5%)*	
48	*APC + RET*<br>*1 (0.4%)*	*BRCA1 + RET*<br>*1 (0.7%)*		*BRAF + MTOR*<br>*1 (0.6%)*	*ATM + RAF1*<br>*1 (0.5%)*	
49	*AR + RET*<br>*1 (0.4%)*	*BRCA2 + CHEK2*<br>*1 (0.7%)*		*BRCA1 + NF1*<br>*1 (0.6%)*	*ATM + RET*<br>*1 (0.5%)*	
50	*AR + TP53*<br>*1 (0.4%)*	*BRCA2 + EGFR*<br>*1 (0.7%)*		*BRCA1 + PTEN*<br>*1 (0.6%)*	*ATM + SMAD4*<br>*1 (0.5%)*	
51	*ATM + CTNNB1*<br>*1 (0.4%)*	*BRCA2 + KRAS*<br>*1 (0.7%)*		*BRCA1 + RET*<br>*1 (0.6%)*	*ATM + TP53*<br>*1 (0.5%)*	
52	*ATM + EGFR*<br>*1 (0.4%)*	*BRCA2 + MAP2K1*<br>*1 (0.7%)*		*BRCA2 + CHEK2*<br>*1 (0.6%)*	*BARD1 + BRCA1*<br>*1 (0.5%)*	
53	*ATM + FBXW7*<br>*1 (0.4%)*	*BRCA2 + NF1*<br>*1 (0.7%)*		*BRCA2 + EGFR*<br>*1 (0.6%)*	*BARD1 + TP53*<br>*1 (0.5%)*	
54	*ATM + PDGFRA*<br>*1 (0.4%)*	*BRCA2 + PIK3CA*<br>*1 (0.7%)*		*BRCA2 + MAP2K1*<br>*1 (0.6%)*	*BRAF + KRAS*<br>*1 (0.5%)*	
55	*ATM + PIK3CA*<br>*1 (0.4%)*	*BRCA2 + PTEN*<br>*1 (0.7%)*		*BRCA2 + NF1*<br>*1 (0.6%)*	*BRAF + PTEN*<br>*1 (0.5%)*	
56	*ATM + RAF1*<br>*1 (0.4%)*	*BRCA2 + RET*<br>*1 (0.7%)*		*BRCA2 + PIK3CA*<br>*1 (0.6%)*	*BRCA1 + NF1*<br>*1 (0.5%)*	
57	*ATM + RET*<br>*1 (0.4%)*	*BRCA2 + TP53*<br>*1 (0.7%)*		*BRCA2 + PTEN*<br>*1 (0.6%)*	*BRCA1 + PTEN*<br>*1 (0.5%)*	
58	*ATM + SMAD4*<br>*1 (0.4%)*	*CHEK2 + EGFR*<br>*1 (0.7%)*		*BRCA2 + RET*<br>*1 (0.6%)*	*BRCA1 + RET*<br>*1 (0.5%)*	
59	*BARD1 + BRCA1*<br>*1 (0.4%)*	*CHEK2 + NF1*<br>*1 (0.7%)*		*BRCA2 + TP53*<br>*1 (0.6%)*	*BRCA2 + CHEK2*<br>*1 (0.5%)*	
60	*BARD1 + TP53*<br>*1 (0.4%)*	*CHEK2 + TP53*<br>*1 (0.7%)*		*CHEK2 + EGFR*<br>*1 (0.6%)*	*BRCA2 + EGFR*<br>*1 (0.5%)*	
61	*BRAF + KRAS*<br>*1 (0.4%)*	*CTNNB1 + KRAS*<br>*1 (0.7%)*		*CHEK2 + NF1*<br>*1 (0.6%)*	*BRCA2 + NF1*<br>*1 (0.5%)*	
62	*BRAF + MTOR*<br>*1 (0.4%)*	*CTNNB1 + PTEN*<br>*1 (0.7%)*		*CHEK2 + TP53*<br>*1 (0.6%)*	*BRCA2 + PIK3CA*<br>*1 (0.5%)*	
63	*BRCA1 + NF1*<br>*1 (0.4%)*	*EGFR + KRAS*<br>*1 (0.7%)*		*CTNNB1 + KRAS*<br>*1 (0.6%)*	*BRCA2 + PTEN*<br>*1 (0.5%)*	
64	*BRCA1 + PTEN*<br>*1 (0.4%)*	*EGFR + MAP2K1*<br>*1 (0.7%)*		*CTNNB1 + PTEN*<br>*1 (0.6%)*	*BRCA2 + RET*<br>*1 (0.5%)*	
65	*BRCA1 + RET*<br>*1 (0.4%)*	*EGFR + PTEN*<br>*1 (0.7%)*		*EGFR + KRAS*<br>*1 (0.6%)*	*BRCA2 + TP53*<br>*1 (0.5%)*	
66	*BRCA2 + CHEK2*<br>*1 (0.4%)*	*EGFR + SMAD4*<br>*1 (0.7%)*		*EGFR + MAP2K1*<br>*1 (0.6%)*	*BRIP1 + PIK3CA*<br>*1 (0.5%)*	
67	*BRCA2 + FBXW7*<br>*1 (0.4%)*	*ERBB2 + MRE11A*<br>*1 (0.7%)*		*EGFR + PTEN*<br>*1 (0.6%)*	*BRIP1 + TP53*<br>*1 (0.5%)*	
68	*BRCA2 + MAP2K1*<br>*1 (0.4%)*	*ERBB2 + STK11*<br>*1 (0.7%)*		*EGFR + SMAD4*<br>*1 (0.6%)*	*CHEK2 + KRAS*<br>*1 (0.5%)*	
69	*BRCA2 + NF1*<br>*1 (0.4%)*	*ERBB2 + TERT*<br>*1 (0.7%)*		*ERBB2 + KRAS*<br>*1 (0.6%)*	*CHEK2 + NF1*<br>*1 (0.5%)*	
70	*BRCA2 + PIK3CA*<br>*1 (0.4%)*	*KRAS + NBN*<br>*1 (0.7%)*		*ERBB2 + MRE11A*<br>*1 (0.6%)*	*CHEK2 + TP53*<br>*1 (0.5%)*	
71	*BRCA2 + PTEN*<br>*1 (0.4%)*	*KRAS + PDGFRA*<br>*1 (0.7%)*		*ERBB2 + STK11*<br>*1 (0.6%)*	*EGFR + KRAS*<br>*1 (0.5%)*	
72	*BRCA2 + RET*<br>*1 (0.4%)*	*KRAS + PIK3CA*<br>*1 (0.7%)*		*ERBB2 + TERT*<br>*1 (0.6%)*	*EGFR + SMAD4*<br>*1 (0.5%)*	
73	*BRIP1 + PIK3CA*<br>*1 (0.4%)*	*KRAS + RET*<br>*1 (0.7%)*		*KRAS + NBN*<br>*1 (0.6%)*	*ERBB2 + KRAS*<br>*1 (0.5%)*	
74	*BRIP1 + TP53*<br>*1 (0.4%)*	*KRAS + SMAD4*<br>*1 (0.7%)*		*KRAS + PDGFRA*<br>*1 (0.6%)*	*ERBB2 + MRE11A*<br>*1 (0.5%)*	

Rank[1]	**Overall (n = 267)**, n (%)[2]	**LUAD (n = 147)**, n (%)[2]	**LUSC (n = 98)**, n (%)[2]	**NSQ (n = 169)**, n (%)[2]	**Male (n = 221)**, n (%)[2]	**Female (n = 46)**, n (%)[2]
75	*CHEK2 + EGFR*<br>*1 (0.4%)*	*MAP2K1 + TP53*<br>*1 (0.7%)*		*KRAS + PIK3CA*<br>*1 (0.6%)*	*ERBB2 + STK11*<br>*1 (0.5%)*	
76	*CHEK2 + NF1*<br>*1 (0.4%)*	*MLH1 + TP53*<br>*1 (0.7%)*		*KRAS + RET*<br>*1 (0.6%)*	*ERBB2 + TERT*<br>*1 (0.5%)*	
77	*CHEK2 + TP53*<br>*1 (0.4%)*	*MRE11A + TP53*<br>*1 (0.7%)*		*KRAS + SMAD4*<br>*1 (0.6%)*	*FBXW7 + NF1*<br>*1 (0.5%)*	
78	*CTNNB1 + KRAS*<br>*1 (0.4%)*	*MTOR + PTEN*<br>*1 (0.7%)*		*MAP2K1 + TP53*<br>*1 (0.6%)*	*FBXW7 + PTEN*<br>*1 (0.5%)*	
79	*CTNNB1 + PTEN*<br>*1 (0.4%)*	*NBN + TP53*<br>*1 (0.7%)*		*MLH1 + TP53*<br>*1 (0.6%)*	*KIT + TP53*<br>*1 (0.5%)*	
80	*EGFR + KRAS*<br>*1 (0.4%)*	*NF1 + PDGFRA*<br>*1 (0.7%)*		*MRE11A + TP53*<br>*1 (0.6%)*	*KRAS + NBN*<br>*1 (0.5%)*	
81	*EGFR + MAP2K1*<br>*1 (0.4%)*	*NF1 + PIK3CA*<br>*1 (0.7%)*		*MTOR + PTEN*<br>*1 (0.6%)*	*KRAS + PDGFRA*<br>*1 (0.5%)*	
82	*EGFR + PTEN*<br>*1 (0.4%)*	*PDGFRA + PIK3CA*<br>*1 (0.7%)*		*NBN + NF1*<br>*1 (0.6%)*	*KRAS + PIK3CA*<br>*1 (0.5%)*	
83	*EGFR + SMAD4*<br>*1 (0.4%)*	*PDGFRA + TP53*<br>*1 (0.7%)*		*NBN + SMAD4*<br>*1 (0.6%)*	*KRAS + RET*<br>*1 (0.5%)*	
84	*ERBB2 + KRAS*<br>*1 (0.4%)*	*PIK3CA + PTEN*<br>*1 (0.7%)*		*NF1 + PDGFRA*<br>*1 (0.6%)*	*KRAS + SMAD4*<br>*1 (0.5%)*	
85	*ERBB2 + MRE11A*<br>*1 (0.4%)*	*PIK3CA + RET*<br>*1 (0.7%)*		*NF1 + PIK3CA*<br>*1 (0.6%)*	*MLH1 + TP53*<br>*1 (0.5%)*	
86	*ERBB2 + STK11*<br>*1 (0.4%)*	*POLE + TP53*<br>*1 (0.7%)*		*NF1 + SMAD4*<br>*1 (0.6%)*	*MRE11A + TP53*<br>*1 (0.5%)*	
87	*ERBB2 + TERT*<br>*1 (0.4%)*	*SMAD4 + TP53*<br>*1 (0.7%)*		*PDGFRA + PIK3CA*<br>*1 (0.6%)*	*MSH6 + PIK3CA*<br>*1 (0.5%)*	
88	*FBXW7 + NF1*<br>*1 (0.4%)*	*TERT + TP53*<br>*1 (0.7%)*		*PDGFRA + TP53*<br>*1 (0.6%)*	*MSH6 + TERT*<br>*1 (0.5%)*	
89	*FBXW7 + PTEN*<br>*1 (0.4%)*			*PIK3CA + PTEN*<br>*1 (0.6%)*	*NBN + NF1*<br>*1 (0.5%)*	
90	*KIT + TP53*<br>*1 (0.4%)*			*PIK3CA + RET*<br>*1 (0.6%)*	*NBN + SMAD4*<br>*1 (0.5%)*	
91	*KRAS + NBN*<br>*1 (0.4%)*			*POLE + TP53*<br>*1 (0.6%)*	*NF1 + PDGFRA*<br>*1 (0.5%)*	
92	*KRAS + PDGFRA*<br>*1 (0.4%)*			*TERT + TP53*<br>*1 (0.6%)*	*NF1 + PIK3CA*<br>*1 (0.5%)*	
93	*KRAS + PIK3CA*<br>*1 (0.4%)*				*NF1 + SMAD4*<br>*1 (0.5%)*	
94	*KRAS + RET*<br>*1 (0.4%)*				*NRAS + TP53*<br>*1 (0.5%)*	
95	*KRAS + SMAD4*<br>*1 (0.4%)*				*PDGFRA + PIK3CA*<br>*1 (0.5%)*	
96	*MAP2K1 + TP53*<br>*1 (0.4%)*				*PDGFRA + TP53*<br>*1 (0.5%)*	
97	*MLH1 + TP53*<br>*1 (0.4%)*				*PIK3CA + RET*<br>*1 (0.5%)*	
98	*MRE11A + TP53*<br>*1 (0.4%)*				*PIK3CA + STK11*<br>*1 (0.5%)*	
99	*MSH6 + PIK3CA*<br>*1 (0.4%)*				*PIK3CA + TERT*<br>*1 (0.5%)*	
100	*MSH6 + TERT*<br>*1 (0.4%)*				*POLE + TP53*<br>*1 (0.5%)*	
101	*MTOR + PTEN*<br>*1 (0.4%)*					
102	*NBN + NF1*<br>*1 (0.4%)*					
103	*NBN + SMAD4*<br>*1 (0.4%)*					
104	*NF1 + PDGFRA*<br>*1 (0.4%)*					
105	*NF1 + PIK3CA*<br>*1 (0.4%)*					
106	*NF1 + SMAD4*<br>*1 (0.4%)*					
107	*NRAS + TP53*<br>*1 (0.4%)*					
108	*PDGFRA + PIK3CA*<br>*1 (0.4%)*					
109	*PDGFRA + TP53*<br>*1 (0.4%)*					
110	*PIK3CA + RET*<br>*1 (0.4%)*					
111	*PIK3CA + STK11*<br>*1 (0.4%)*					
112	*PIK3CA + TERT*<br>*1 (0.4%)*					

Rank[1]	**Overall (n = 267)**, n (%)[2]	**LUAD (n = 147)**, n (%)[2]	**LUSC (n = 98)**, n (%)[2]	**NSQ (n = 169)**, n (%)[2]	**Male (n = 221)**, n (%)[2]	**Female (n = 46)**, n (%)[2]
113	*POLE + TP53*<br>*1 (0.4%)*					

[1] Pairs ranked frequency-wise independently within each subgroup by number of patients with detected co-mutations.
[2] Percentages (%) for recorded cases are calculated within columns.

Table S8. PD-L1 and TIL expression by patient & tumour characteristics, and by biomarkers, n=367.

		PD-L1 TPS (%)				TILs (%)			
		PD-L1 <1% n (%)[1]	PD-L1 ≥1% n (%)[1]	NE[4] n (%)[1]	*p* value[2]	TILs <10% n (%)[1]	TILs ≥10% n (%)[1]	NE[4] n (%)[1]	*p* value[2]
Patient Characteristics	**Age**				0.093$^{\chi2}$				0.413$^{\chi2}$
	<65 years	110 (46.8%)	37 (58.7%)	31 (44.9%)		45 (45.9%)	129 (50.8%)	4 (26.7%)	
	≥65 years	125 (53.2%)	26 (41.3%)	38 (55.1%)		53 (54.1%)	125 (49.2%)	11 (73.3%)	
	**Sex**				0.062$^{\chi2}$				0.785$^{\chi2}$
	Male	189 (80.4%)	57 (90.5%)	56 (81.2%)		81 (82.7%)	213 (83.9%)	8 (53.3%)	
	Female	46 (19.6%)	6 (9.5%)	13 (18.8%)		17 (17.3%)	41 (16.1%)	7 (46.7%)	
	**Smoking status**				0.056$^{\chi2}$				0.845$^{\chi2}$
	Non-smoker	26 (11.3%)	2 (3.2%)	7 (10.4%)		9 (9.5%)	22 (8.8%)	4 (26.7%)	
	Smoker	205 (88.7%)	60 (96.8%)	60 (89.6%)		86 (90.5%)	228 (91.2%)	11 (73.3%)	
	NA[3]	4 (1.7%)	1 (1.6%)	2 (2.9%)		3 (3.1%)	4 (1.6%)	0 (0%)	
	**ECOG PS**				0.742^{FET}				0.265^{FET}
	0-1	223 (94.9%)	61 (96.8%)	64 (92.8%)		91 (92.9%)	244 (96.1%)	13 (86.7%)	
	≥2	12 (5.1%)	2 (3.2%)	5 (7.2%)		7 (7.1%)	10 (3.9%)	2 (13.3%)	
Tumour Characteristics	**Histology**				0.318$^{\chi2}$				0.418$^{\chi2}$
	NSQ	154 (65.5%)	37 (58.7%)	47 (68.1%)		67 (68.4%)	162 (63.8%)	9 (60%)	
	LUSC	81 (34.5%)	26 (41.3%)	22 (31.9%)		31 (31.6%)	92 (36.2%)	6 (40%)	
	**Grade**				**0.032**$^{\chi2}$				0.287$^{\chi2}$
	1-2	91 (45.3%)	15 (28.8%)	18 (36%)		28 (36.4%)	94 (43.3%)	2 (22.2%)	
	3-4	110 (54.7%)	37 (71.2%)	32 (64%)		49 (63.6%)	123 (56.7%)	7 (77.8%)	
	NA	34 (14.5%)	11 (17.5%)	19 (27.5%)		21 (21.4%)	37 (14.6%)	6 (40%)	
	**Stage**[5]				0.644$^{\chi2}$				**<0.001**$^{\chi2}$
	I-III	148 (63.5%)	42 (66.7%)	26 (37.7%)		40 (41.2%)	172 (68%)	4 (26.7%)	
	IV	85 (36.5%)	21 (33.3%)	43 (62.3%)		57 (58.8%)	81 (32%)	11 (73.3%)	
	NA[3]	2 (0.9%)	0 (0%)	0 (0%)		1 (1%)	1 (0.4%)	0 (0%)	
Biomarkers	**TILs (%)**				**0.040**$^{\chi2}$				
	<10%	67 (29.5%)	10 (16.4%)	21 (32.8%)					
	≥10%	160 (70.5%)	51 (83.6%)	43 (67.2%)					
	NE[4]	8 (3.4%)	2 (3.2%)	5 (7.2%)					
	**Gene PV status**				0.788$^{\chi2}$				0.327$^{\chi2}$
	Wild type	32 (16.4%)	9 (18%)	8 (36.4%)		10 (14.7%)	38 (20.1%)	1 (10%)	
	Mutated	163 (83.6%)	41 (82%)	14 (63.6%)		58 (85.3%)	151 (79.9%)	9 (90%)	
	NE[4]	40 (17%)	13 (20.6%)	47 (68.1%)		30 (30.6%)	65 (25.6%)	5 (33.3%)	
	***TP53* status**				0.599$^{\chi2}$				0.155$^{\chi2}$
	Wild type	90 (46.2%)	21 (42%)	13 (59.1%)		27 (39.7%)	94 (49.7%)	3 (20%)	
	Mutated	105 (53.8%)	29 (58%)	9 (40.9%)		41 (60.3%)	95 (50.3%)	7 (46.7%)	
	NE	40 (17%)	13 (20.6%)	47 (68.1%)		30 (30.6%)	65 (25.6%)	5 (33.3%)	
	**Co-mutation status**				0.332$^{\chi2}$				0.561$^{\chi2}$
	<2 mutated genes	114 (58.5%)	33 (66.0%)	13 (59.1%)		39 (57.4%)	116 (61.4%)	5 (50%)	
	≥2 mutated genes	81 (41.5%)	17 (34.0%)	9 (40.9%)		29 (42.6%)	73 (38.6%)	5 (50%)	
	NE[4]	40 (17%)	13 (20.6%)	47 (68.1%)		30 (30.6%)	65 (25.6%)	5 (33.3%)	
	**SCNA status**				0.708$^{\chi2}$				**0.034**$^{\chi2}$
	No SCNA	153 (78.5%)	38 (76%)	16 (72.7%)		47 (69.1%)	154 (81.5%)	6 (60%)	
	SCNA-positive	42 (21.5%)	12 (24%)	6 (27.3%)		21 (30.9%)	35 (18.5%)	4 (40%)	
	NE[4]	40 (17%)	13 (20.6%)	47 (68.1%)		30 (30.6%)	65 (25.6%)	5 (33.3%)	

Statistics for PD-L1 (%) and TILs (%) crosstabulations

Variable	PD-L1 (<1%/ ≥1%)				TILs (<10%/ ≥10%)			
	n	*p* value	Test & value	OR (95% CI)	n	*p* value	Test & value	OR (95% CI)
**Age (<65/ ≥65 years)**	298	0.093	χ^2; $\chi2(1)$=2.825	0.62 (0.34-1.13)	352	0.413	χ^2; $\chi2(1)$=0.671	0.82 (0.50-1.35)
**Sex (Male/ Female)**	298	0.062	χ^2; $\chi2(1)$=3.484	0.43 (0.14-1.09)	352	0.785	χ^2; $\chi2(1)$=0.075	0.92 (0.48-1.82)
**Smoking status (Non-smoker/ Smoker)**	293	0.056	χ^2; $\chi2(1)$=3.646	3.79 (0.91-33.9)	345	0.845	χ^2; $\chi2(1)$=0.038	1.08 (0.42-2.57)
**ECOG PS (0-1/ ≥2)**	298	0.742	FET; (-)	0.61 (0.06-2.85)	352	0.265	FET; (-)	0.53 (0.18-1.71)
**Histology (NSQ/ LUSC)**	298	0.318	χ^2; $\chi2(1)$=0.999	1.33 (0.72-2.44)	352	0.418	χ^2; $\chi2(1)$=0.655	1.23 (0.73-2.09)
**Grade (G1-2/ G3-4)**	253	**0.032**	**χ^2; $\chi2(1)$=4.580**	**2.04 (1.01-4.26)**	294	0.287	χ^2; $\chi2(1)$=1.132	0.75 (0.42-1.32)
**Stage (I-III/ IV)**	296	0.644	χ^2; $\chi2(1)$=0.214	0.87 (0.46-1.62)	350	**<0.001**	**χ^2; $\chi^2(1)$=21.004**	**0.33 (0.20-0.55)**
**TILs (<10%/ ≥10%)**	288	**0.040**	**χ^2; $\chi2(1)$=4.226**	**2.13 (0.99-4.99)**				
**Gene PV status (Wild type/ Mutated)**	245	0.788	χ^2; $\chi2(1)$=0.072	0.89 (0.38-2.30)	257	0.327	χ^2; $\chi2(1)$=0.096	0.69 (0.29-1.52)
***TP53* status (Wild type/ Mutated)**	245	0.599	χ^2; $\chi2(1)$=0.277	1.18 (0.63-2.22)	257	0.155	χ^2; $\chi2(1)$=2.019	0.67 (0.38-1.17)
**Comutation status (<2/ ≥2 mut, genes)**	245	0.332	χ^2; $\chi2(1)$=0.942	0.73 (0.35-1.45)	257	0.561	χ^2; $\chi2(1)$=0.338	0.85 (0.47-1.55)
**SCNA status (Np SCNA/ SCNA positive)**	245	0.708	χ^2; $\chi2(1)$=0.140	1.15 (0.50-2.50)	257	**0.034**	**χ^2; $\chi^2(1)$=4.486**	**0.51 (0.26-1.02)**

(1) Percentages (%) for recorded cases use valid denominators and are calculated within columns. NA percentages (%) use total denominators, n=367. Valid percentages may not sum to exactly 100% due to one decimal rounding.
(2) Statistical testing performed excluding NA & NE. FET: Fisher's exact test, χ^2: Pearson's Chi-squared test.
(3) NA corresponds to not available/ missing data.
(4) NE corresponds to patients whose tissue was not tested.
(5) Stage refers to either clinical or pathological stage at diagnosis.

Table S9. TIL density and PD-L1 expression analysis restricted to patients with both biomarkers evaluated (n=288).

TIL expression		PD-L1 expression						
**TILs (%) Variable**	**All**, n=288	**PD-L1 TPS (<1/ ≥1%)**, n (%)[(1)]			**PD-L1 TPS (<1%/ 1-50%/ ≥50%)**, n (%)[(1)]			
		**<1%**, n=227	**≥1%**, n=61	***p* value**	**<1%**, n=227	**[1-49%)**, n=44	**≥50%**, n=17	***p* value**
**TILs continuous (%)**				**0.011**^{KW}				**0.036**^{KW}
Mean ±SD	35.79±30.55	33.50±30.15	44.33±30.77		33.50±30.15	45.86±30.76	40.35±31.37	
Median [Q1, Q3]	20 [5, 70]	20 [5, 60]	50 [10, 80]		20 [5, 60]	55 [18, 80]	30 [10, 70]	
**TILs (%) 10% cut-off**				**0.040**$^{\chi 2}$				0.117$^{\chi 2}$
<10%	77 (26.7%)	67 (29.5%)	10 (16.4%)		67 (29.5%)	8 (18.2%)	2 (11.8%)	
≥10%	211 (73.3%)	160 (70.5%)	51 (83.6%)		160 (70.5%)	36 (81.8%)	15 (88.2%)	
**TILs (%) 50% cut-off**				**0.017**$^{\chi 2}$				**0.031**$^{\chi 2}$
<50%	175 (60.8%)	146 (64.3%)	29 (47.5%)		146 (64.3%)	19 (43.2%)	10 (58.8%)	
≥50%	113 (39.2%)	81 (35.7%)	32 (52.5%)		81 (35.7%)	25 (56.8%)	7 (41.2%)	
**TILs (%) 4 groups**				0.081$^{\chi 2}$				0.134$^{\chi 2}$
<10%	77 (26.7%)	67 (29.5%)	10 (16.4%)		67 (29.5%)	8 (18.2%)	2 (11.8%)	
[10%-25%)	68 (23.6%)	55 (24.2%)	13 (21.3%)		55 (24.2%)	7 (15.9%)	6 (35.3%)	
[25%-50%)	30 (10.4%)	24 (10.6%)	6 (9.8%)		24 (10.6%)	4 (9.1%)	2 (11.8%)	
≥50%	113 (39.2%)	81 (35.7%)	32 (52.5%)		81 (35.7%)	25 (56.8%)	7 (41.2%)	

**Spearman's rank correlation coefficient**			
**Variables**	**n**	**r_s**	***p* value**
PD-L1 TPS (%) vs TILs (%)	288	0.146	**0.013**

[(1)] Percentages are calculated within columns.

Table S10. Treatment pathways and administered drugs, n = 367.

Characteristic	**Overall**, n (%)
**Surgical resection**	
**Surgery**	n = 367 (100%)
No	152 (41.4%)
Yes	215 (58.6%)
**Surgery type**	n = 211 (57.5%)
Lobectomy	151 (70.2%)
Other	6 (2.8%)
Pneumonectomy	38 (17.7%)
Segmentectomy	16 (7.6%)
Missing	4 (1.1%)
Not applicable	156 (42.5%)
**SACT**	
**Treatment Pathways**	n = 237 (64.6%)
1st Line	83 (35.0%)
1st Line, 2nd Line	60 (25.3%)
1st Line, 2nd Line, 3rd Line	24 (10.1%)
1st Line, 2nd Line, 3rd Line, 4th Line	1 (0.4%)
1st Line, 2nd Line, 3rd Line, 4th Line, 5th Line	1 (0.4%)
1st Line, 2nd Line, 3rd Line, 4th Line, 5th Line, 6th Line	2 (0.8%)
1st Line, 2nd Line, 3rd Line, 4th Line, 5th Line, 6th Line, 7th Line	1 (0.4%)
2nd Line	1 (0.4%)
2nd Line, 3rd Line	1 (0.4%)
3rd Line	1 (0.4%)
Adjuvant	29 (12.2%)
Adjuvant, 1st Line	10 (4.2%)
Adjuvant, 1st Line, 2nd Line	7 (3.0%)
Adjuvant, 1st Line, 2nd Line, 3rd Line	4 (1.7%)
Adjuvant, 1st Line, 2nd Line, 3rd Line, 4th Line	3 (1.3%)
Adjuvant, 1st Line, 2nd Line, 3rd Line, 4th Line, 5th Line	1 (0.4%)
Adjuvant, 1st Line, 2nd Line, 3rd Line, 4th Line, 5th Line, 6th Line	1 (0.4%)
Adjuvant, 2nd Line	1 (0.4%)
Neoadjuvant, 1st Line	2 (0.8%)
Neoadjuvant, 1st Line, 2nd Line	2 (0.8%)
Neoadjuvant, Adjuvant, 2nd Line	1 (0.4%)
Neoadjuvant, Adjuvant, 2nd Line, 3rd Line, 4th Line	1 (0.4%)
Not applicable	130 (35.4%)
**SACT lines**	
**Neoadjuvant**	n = 237 (64.6%)
No	231 (97.5%)
Yes	6 (2.5%)
Not applicable	130 (35.4%)
**Adjuvant**	n = 237 (64.6%)
No	179 (75.5%)
Yes	58 (24.5%)
Not applicable	130 (35.4%)
**1st Line**	n = 237 (64.6%)
No	35 (14.8%)
Yes	202 (85.2%)
Not applicable	130 (35.4%)
**2nd Line**	n = 237 (64.6%)
No	125 (52.7%)
Yes	112 (47.3%)
Not applicable	130 (35.4%)
**3rd Line**	n = 237 (64.6%)
No	196 (82.7%)
Yes	41 (17.3%)
Not applicable	130 (35.4%)
**4th Line**	n = 237 (64.6%)
No	226 (95.4%)
Yes	11 (4.6%)
Not applicable	130 (35.4%)
**5th Line**	n = 237 (64.6%)
No	231 (97.5%)
Yes	6 (2.5%)
Not applicable	130 (35.4%)
**6th Line**	n = 237 (64.6%)
No	233 (98.3%)
Yes	4 (1.7%)
Not applicable	130 (35.4%)
**7th Line**	n = 237 (64.6%)
No	236 (99.6%)
Yes	1 (0.4%)
Not applicable	130 (35.4%)
**SACT drugs**	
**Paclitaxel**	n = 237 (64.6%)
No	171 (72.2%)
Yes	66 (27.8%)
Not applicable	130 (35.4%)
**Carboplatin**	n = 237 (64.6%)
No	41 (17.3%)
Yes	196 (82.7%)
Not applicable	130 (35.4%)
**Gemcitabine**	n = 237 (64.6%)

Characteristic	Overall, n (%)
No	169 (71.3%)
Yes	68 (28.7%)
Not applicable	130 (35.4%)
**Docetaxel**	n = 237 (64.6%)
No	144 (60.8%)
Yes	93 (39.2%)
Not applicable	130 (35.4%)
**Pemetrexed**	n = 237 (64.6%)
No	179 (75.5%)
Yes	58 (24.5%)
Not applicable	130 (35.4%)
**Vinorelbine**	n = 237 (64.6%)
No	211 (89.0%)
Yes	26 (11.0%)
Not applicable	130 (35.4%)
**Gefitinib**	n = 237 (64.6%)
No	224 (94.5%)
Yes	13 (5.5%)
Not applicable	130 (35.4%)
**Cisplatin**	n = 237 (64.6%)
No	203 (85.7%)
Yes	34 (14.3%)
Not applicable	130 (35.4%)
**Erlotinib**	n = 237 (64.6%)
No	219 (92.4%)
Yes	18 (7.6%)
Not applicable	130 (35.4%)
**Etoposide**	n = 237 (64.6%)
No	232 (97.9%)
Yes	5 (2.1%)
Not applicable	130 (35.4%)
**Bevacizumab**	n = 237 (64.6%)
No	207 (87.3%)
Yes	30 (12.7%)
Not applicable	130 (35.4%)
**Lapatinib**	n = 237 (64.6%)
No	231 (97.5%)
Yes	6 (2.5%)
Not applicable	130 (35.4%)
**Nivolumab**	n = 237 (64.6%)
No	191 (80.6%)
Yes	46 (19.4%)
Not applicable	130 (35.4%)
**Vinorelbine oral**	n = 237 (64.6%)
No	228 (96.2%)
Yes	9 (3.8%)
Not applicable	130 (35.4%)
**Cabazitaxel**	n = 237 (64.6%)
No	236 (99.6%)
Yes	1 (0.4%)
Not applicable	130 (35.4%)
**Nab-Paclitaxel**	n = 237 (64.6%)
No	231 (97.5%)
Yes	6 (2.5%)
Not applicable	130 (35.4%)
**Afatinib**	n = 237 (64.6%)
No	233 (98.3%)
Yes	4 (1.7%)
Not applicable	130 (35.4%)
**Osimertinib**	n = 237 (64.6%)
No	235 (99.2%)
Yes	2 (0.8%)
Not applicable	130 (35.4%)
**Pembrolizumab**	n = 237 (64.6%)
No	231 (97.5%)
Yes	6 (2.5%)
Not applicable	130 (35.4%)
**Durvalumab**	n = 237 (64.6%)
No	235 (99.2%)
Yes	2 (0.8%)
Not applicable	130 (35.4%)
**Atezolizumab**	n = 237 (64.6%)
No	233 (98.3%)
Yes	4 (1.7%)
Not applicable	130 (35.4%)
**Temozolomide**	n = 237 (64.6%)
No	235 (99.2%)
Yes	2 (0.8%)
Not applicable	130 (35.4%)
**Bavituximab**	n = 237 (64.6%)
No	235 (99.2%)
Yes	2 (0.8%)
Not applicable	130 (35.4%)
**Crizotinib**	n = 237 (64.6%)
No	235 (99.2%)
Yes	2 (0.8%)

Characteristic	Overall, n (%)
Not applicable	130 (35.4%)
**Nintedanib**	n = 237 (64.6%)
No	235 (99.2%)
Yes	2 (0.8%)
Missing	130 (35.4%)
**Cabozantinib**	n = 237 (64.6%)
No	236 (99.6%)
Yes	1 (0.4%)
Not applicable	130 (35.4%)
**Zoledronic Acid**	n = 237 (64.6%)
No	236 (99.6%)
Yes	1 (0.4%)
Missing	130 (35.4%)
**Ipilimumab**	n = 237 (64.6%)
No	234 (98.7%)
Yes	3 (1.3%)
Not applicable	130 (35.4%)
**IO-based SACT**	
**IO-based SACT**	n = 237 (64.6%)
No	181 (76.4%)
Yes	56 (23.6%)
Not applicable	130 (35.4%)
**TKI-based SACT**	
**TKI-based SACT**	n = 237 (64.6%)
No	195 (82.3%)
Yes	42 (17.7%)
Not applicable	130 (35.4%)

Table S11. Follow up and overall survival statistics, n = 367[(1)].

Follow up Statistics			
**Follow up (months)**		**Survival status**	
**Mean (SD)**	**51.3 (53.4)**	Alive	60 (16.3%)
**Median (95% CI)**	**29.5 (25.4–33.9)**	Dead	306 (83.4%)
Min, Max	0, 230	NA[(2)]	1 (0.3%)
IQR	12.6 – 74.5		
NA[(2)]	1		

Overall survival Statistics

Overall survival (months)						
**Patient subset**	**n**	**Mean (SD)**	**Median [Min, Max]**	**IQR**	**KM Median (95% CI)**	**NA[(2)]**
Whole cohort	367	51.3 (53.4)	29.5 [0.010, 230]	12.6 – 74.5	29.5 (25.4–35.0)	1
Surgical cohort	215	70.8 (58.2)	47.0 [0.010, 230]	23.1 – 113.3	50.3 (40.5–74.5)	1
1st line SACT cohort	202	25.2 (27.4)	15.1 [0.033, 145]	7.3 – 31.2	15.1 (13.2–18.9)	1

(1) Survival outcome data available for n = 366.
(2) NA corresponds to missing/ not available data.

Table S12. Univariable overall survival analysis for individual co-mutation pairs detected in ≥2% of sequenced tumours, n=267.

Co-mutation pair[(1)]	N (%)[(2)]	HR (95% CI)[(3)]	*p* value[(4)]
TP53/PTEN	15 (5.6%)	1.01 (0.56-1.81)	0.968
STK11/KRAS	13 (4.9%)	1.63 (0.91-2.93)	0.097
TP53/KRAS	12 (4.5%)	0.73 (0.36-1.49)	0.391
TP53/EGFR	10 (3.7%)	1.08 (0.55-2.1)	0.826
PIK3CA/PTEN	7 (2.6%)	1.21 (0.57-2.57)	0.625
NF1/PTEN	6 (2.2%)	0.86 (0.32-2.3)	0.758
KRAS/PTEN	6 (2.2%)	0.81 (0.3-2.19)	0.681

[(1)] Each pair was compared against all other sequenced tumours.

[(2)] Percentages are calculated using the 267 sequenced tumours as denominators.

[(3)] Hazard ratios are from univariable Cox regression.

[(4)] *p* values are from the log-rank test.

Table S13. Overall survival among *TP53*-mutant tumours by number of *TP53* pathogenic variants and by co-mutation status.

			Unadjusted		Adjusted	
Comparison[(1)]	n (deaths)	Median OS, months (95% CI)	HR (95% CI)[(2)]	*p*	HR (95% CI)	*p*
Variant level: one vs two pathogenic variants within *TP53* (*TP53*-mutant tumours)						
One *TP53* PV	138 (111)	32.4 (26.2–44.2)	Reference		Reference	
Two *TP53* PVs[(3)]	5 (5)	33.8 (5.8–NR)	1.47 (0.60–3.61)	0.4000	1.46 (0.59–3.63)	0.4099
Gene level: *TP53* alone vs *TP53* with a co-mutation (*TP53*-mutant tumours)						
TP53 only	97 (78)	30.8 (25.4–46.7)	Reference		Reference	
TP53 + co-mutation	46 (38)	38.0 (21.9–55.7)	1.01 (0.68–1.49)	0.9711	1.08 (0.72–1.62)	0.6984
Three-level comparison, all sequenced tumours						
TP53 wild-type	124 (103)	30.6 (23.0–43.5)	Reference		Reference	
TP53 only	97 (78)	30.8 (25.4–46.7)	0.97 (0.72–1.30)	0.8250	1.02 (0.75–1.39)	0.8773
TP53 + co-mutation	46 (38)	38.0 (21.9–55.7)	1.00 (0.69–1.45)	0.9874	1.12 (0.76–1.64)	0.5718

(1) Analyses are restricted to the 267 sequenced tumours. Gene-level mutation status counts a gene as altered whether one or two pathogenic variants were detected, as throughout the study.

(2) Hazard ratios are from Cox regression; adjusted models include age (≥65 vs <65 years), sex, smoking status, histology and stage (I–III vs IV), and are complete-case.

(3) Only five tumours carried two distinct TP53 pathogenic variants; that comparison is descriptive and is not interpreted.

Table S14. Sensitivity analysis – Stratified Cox Regression (Age <65 years/ ≥65 years).[(1)]

	Main model		Stratified Sensitivity model	
**Factor**	**Main HR (95% CI)**	***p* value**	**Stratified HR (95% CI)**	***p* value**
Patient characteristics				
**Age: ≥65 years** (vs <65 years)	**1.42 (1.06–1.90)**	**0.020**		
**Sex: Male** (vs Female)	0.94 (0.62-1.45)	0.794	0.93 (0.61–1.43)	0.746
**Smoking status: Smoker** (vs Non-smoker)	1.00 (0.60–1.66)	0.998	0.97 (0.58–1.62)	0.910
Tumour characteristics				
**Histology: LUSC** (vs LUAD)	0.99 (0.73–1.36)	0.969	1.02 (0.74–1.40)	0.903
**Histology: Other** (vs LUAD)	1.52 (0.92–2.52)	0.102	1.57 (0.95–2.60)	0.080
**Stage II** (vs I)	**1.69 (1.07–2.65)**	**0.024**	**1.66 (1.06–2.63)**	**0.028**
**Stage III** (vs I)	**2.12 (1.33–3.38)**	**0.002**	**2.16 (1.35–3.44)**	**0.001**
**Stage IV** (vs I)	**4.51 (2.97–6.85)**	**<0.001**	**4.58 (3.01–6.97)**	**<0.001**
Microscopy biomarkers				
**TIL expression: TILs ≥10%** (vs <10%)	**0.72 (0.52–0.99)**	**0.042**	**0.71 (0.52–0.98)**	**0.040**
Molecular biomarkers				
**Co-mutation status: ≥2 mutated genes** (vs <2)	**1.44 (1.08–1.92)**	**0.014**	**1.47 (1.10–1.97)**	**0.010**
**SCNA status: SCNA-positive** (vs no SCNA detected)	1.31 (0.93-1.83)	0.121	1.30 (0.93–1.83)	0.128

(1) Complete case analysis, n=254, events=208.

Table S15. Stage-stratified analysis.[(1)]

Biomarker	Stage stratum	n	Events	Median OS, reference (months)	Median OS, index (months)	Log-rank p	Unadjusted HR	*p* value	Adjusted HR	*p* value
TIL density ≥10%	I–III (non-metastatic)	211	159	30.0	67.6	0.004	0.57 (95% CI 0.38–0.84)	0.005	**0.60 (95% CI 0.40–0.89)**	**0.012**
	IV (metastatic)	138	131	9.9	15.2	0.081	0.73 (95% CI 0.51–1.04)	0.082	**0.72 (95% CI 0.50–1.04)**	**0.080**
	Interaction (biomarker × stage)	344	287	—	—	—	—	—	1.05 (0.62–1.80)	0.849
≥2 mutated genes	I–III (non-metastatic)	160	118	68.1	48.7	0.383	1.18 (95% CI 0.82–1.70)	0.383	1.19 (95% CI 0.81–1.74)	0.379
	IV (metastatic)	106	100	15.1	9.6	0.009	1.73 (95% CI 1.14–2.63)	0.01	**1.83 (95% CI 1.19–2.79)**	**0.005**
	Interaction (biomarker × stage)	264	217	—	—	—	—	—	1.51 (0.86–2.62)	0.145

(1) Hazard ratios adjusted for age (≥65 vs <65 years), sex, smoking status and histology. Stage is the stratifying variable and is therefore not a covariate within strata. Interaction rows report the likelihood-ratio test for biomarker × stage on the pooled cohort. Analyses are exploratory.

Table S16. List of genes tested in the Medicover Genetics ForeSENTIA Pan-Cancer Core assay.

Genes tested for molecular alterations		
SNVs, MNVs, Insertions and Deletions	SCNAs	Translocations
AKT1, ALK, APC, AR, ARAF, ATM, ATRX, BARD1, BRAF, BRCA1, BRCA2, BRIP1, CDH1, CHEK2, CIC, CTNNB1, DDR2, DICER1, EGFR, ERBB2, ERBB3, ERBB4, ESR1, FBXW7, FLT3, FOXA1, FOXL2, FUBP1, GATA3, GNA11, GNAQ, GNAS, H3F3A, IDH1, IDH2, JAK2, KEAP1, KIT, KRAS, MAP2K1, MAP3K1, MET, MLH1, MRE11A, MSH2, MSH6, MTOR, NBN, NF1, NPM1, NRAS, NTRK1, PALB2, PDGFRA, PIK3CA, PIK3CB, PMS2, POLE, PTEN, RAD51C, RAD51D, RAF1, RET, RUNX1, SMAD4, SPOP, STK11, TERT, TP53	*AR, CDKN2A, EGFR, ERBB2, ESR1, FGFR1, FGFR2, FGFR3, KIT, KRAS, MET, MYC, MYCN, PIK3CA, PTEN, RB1, TP53*	*ALK, BRAF, FGFR3, NTRK1, NTRK2, NTRK3, RET, ROS1, TMPRSS2*
